# Early serum albumin dynamics in sepsis differ by albumin source and have implications for nutritional assessment

**DOI:** 10.3389/fnut.2026.1858939

**Published:** 2026-07-09

**Authors:** Yifei Ma, Kongyuan Wei, Weiqi Lyu, Yuxuan Tang, Tianyi Zhang, Qingyong Ma, Li Wen, Zheng Wang

**Affiliations:** 1 Department of Hepatobiliary Surgery, The First Affiliated Hospital of Xi'an Jiaotong University, Xi'an, China; 2 Biobank, The First Affiliated Hospital of Xi'an Jiaotong University, Xi'an, China; 3 Pancreatic Diseases Center of Xi'an Jiaotong University, Xi'an, China; 4 Department of General, Visceral and Transplantation Surgery, University of Heidelberg, Heidelberg, Germany; 5 Huawei Technologies Co., Ltd., Xi'an, China; 6 Peking Union Medical College Hospital, Chinese Academy of Medical Sciences and Peking Union Medical College, Beijing, China

**Keywords:** biomarker, human albumin solution, hypoalbuminemia, nutritional assessment, sepsis, serum albumin

## Abstract

**Background:**

Serum albumin is widely used in clinical practice as a nutrition-related biomarker, yet in sepsis its concentration is strongly influenced by inflammation, capillary leak, fluid redistribution, hepatic dysfunction, and exogenous albumin administration. Although hypoalbuminemia is consistently associated with poor outcomes, exogenous human albumin solution (HAS) has not shown a clear survival benefit in septic patients. We therefore investigated whether the prognostic significance of early serum albumin change differs according to whether the change is spontaneous or occurs in the context of HAS administration.

**Methods:**

We analyzed septic patients from the MIMIC-IV database and assessed consistency in an independent external cohort from the First Affiliated Hospital of Xi’an Jiaotong University. Delta albumin (ΔALB) was defined as the change in serum albumin during the first 3 ICU days. Patients were stratified according to HAS use during this period. The primary outcome was 90-day survival.

**Results:**

Among patients who did not receive HAS, higher ΔALB was significantly associated with lower 90-day mortality risk (HR per 1-g/dL increase = 0.50, 95% CI 0.40–0.62, *p* < 0.001), whereas this association was not observed in patients who received HAS. In the external cohort, the absence of a clear prognostic association in albumin-treated patients was directionally consistent, although validation of endogenous ΔALB was limited because most patients received HAS. Endogenous ΔALB was associated with baseline albumin, hematocrit change, platelet change, crystalloid volume, and liver-related variables, suggesting important influences of hemodilution, illness severity, circulatory status, and hepatic dysfunction.

**Conclusion:**

In the primary MIMIC-IV cohort, spontaneous increases in serum albumin, but not albumin changes among HAS-treated patients, were associated with better outcomes. These findings suggest that early albumin recovery should be interpreted cautiously in nutritional assessment, because its clinical meaning depends on albumin source and on non-nutritional physiological factors rather than nutritional status alone.

## Introduction

Serum albumin has long been used in clinical practice as a nutrition-related laboratory marker, and low albumin levels are often interpreted as evidence of malnutrition or protein depletion. However, in critically ill patients, especially those with sepsis, serum albumin is influenced by many non-nutritional processes. Sepsis is a life-threatening syndrome caused by a dysregulated host response to infection and remains a major cause of morbidity and mortality worldwide (1–5). In this setting, systemic inflammation, endothelial dysfunction, vascular leakage, fluid redistribution, hemodilution, altered synthesis and catabolism, and organ dysfunction can all lower measured serum albumin independent of nutritional intake alone.

Hypoalbuminemia is common in sepsis and is consistently associated with poor outcomes (6). Human albumin solution (HAS) is commonly used to increase circulating albumin levels (7), yet randomized trials and guideline statements have not demonstrated a clear survival benefit of albumin administration in septic patients (8–10). This discrepancy is clinically important for nutritional assessment. If serum albumin rises after exogenous replacement without corresponding improvement in the underlying physiological state, then albumin change cannot be interpreted as equivalent to nutritional recovery or therapeutic success. Given the potential risks associated with blood-derived products (11) and the uncertain cost-effectiveness of albumin therapy (12), the biological and clinical meaning of albumin dynamics in sepsis remains controversial.

One possible explanation is that the prognostic significance of albumin change depends on its source, yet prior studies have rarely distinguished spontaneous albumin dynamics from changes occurring after exogenous replacement. If endogenous recovery of serum albumin reflects improvement in vascular integrity, stabilization of fluid balance, resolution of inflammatory stress, or recovery of hepatic synthetic function, its clinical meaning may differ substantially from that of albumin changes observed after HAS administration.

In this study, we examined the relationships among albumin dynamics, HAS exposure, and outcomes in sepsis. Specifically, we distinguished endogenous changes in serum albumin from changes occurring after exogenous albumin administration and evaluated their respective prognostic implications. We further explored clinical variables associated with endogenous albumin change to clarify how early serum albumin dynamics should be interpreted in nutritional assessment of septic patients.

## Materials and methods

### Data sources and study population

The primary cohort was derived from the MIMIC-IV database (version 2.0), which contains detailed information on vital signs, laboratory tests, and in-hospital treatments for 315,460 patients treated in Boston between 2008 and 2019 (13). To assess consistency in an independent clinical setting, we also included an independent retrospective cohort from the Biobank of the First Affiliated Hospital of Xi’an Jiaotong University.

**Figure 1:**
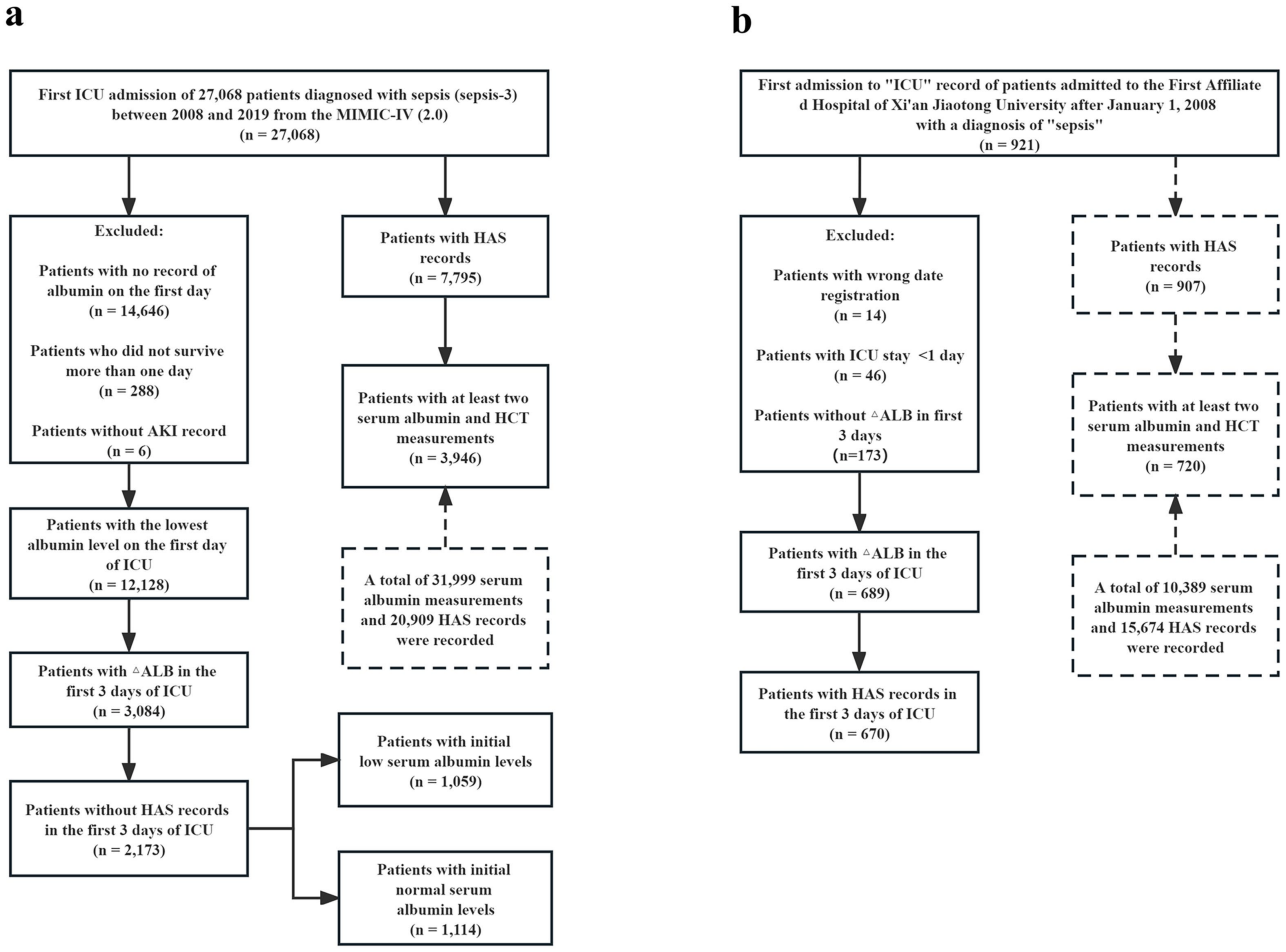
Screening process for the sepsis cohorts. **(a)** MIMIC-IV cohort (version 2.0). **(b)** The First Affiliated Hospital of Xi’an Jiaotong University cohort. ICU, intensive care unit; MIMIC-IV, Medical Information Mart for Intensive Care, version IV; HAS, human albumin solution; ΔALB, change in serum albumin during the first 3 days after ICU admission; HCT, hematocrit; AKI, acute kidney injury.

Sepsis was identified according to the Sepsis-3 criteria, defined as suspected infection with a Sequential Organ Failure Assessment (SOFA) score of 2 or higher, assuming a baseline SOFA score of 0 before admission. ICU admission indexed by stay_id was used as the unit of analysis in MIMIC-IV. For patients with sepsis during more than one ICU stay, only the earliest eligible stay was included. In the Xi’an Jiaotong University cohort, we screened patients admitted to the ICU between 2008 and 2023 whose earliest admission diagnosis included sepsis. In total, 921 patients with sepsis were identified. The screening process for both cohorts is shown in Figures 1a,b.

### Variable definitions and outcomes

Baseline serum albumin was the lowest serum albumin concentration measured on ICU day 1. It was used to evaluate the prognostic association of initial albumin status and to define baseline albumin strata in subsequent subgroup and interaction analyses.

To characterize early albumin dynamics, delta serum albumin (ΔALB) was defined as the change in serum albumin concentration during the first 72 h after ICU admission. ΔALB was calculated in patients with at least two serum albumin measurements during this period, provided that the interval between the earliest and latest measurements was at least 24 h. ΔALB was calculated as the latest value minus the earliest value. Positive values indicated an increase in serum albumin concentration, whereas negative values indicated a decrease. The same approach was used to define changes in other variables analyzed later, including ΔMAP, ΔHCT, ΔPLT, ΔALT, and ΔINR.

In patients who did not receive human albumin solution (HAS) during the first 3 ICU days, ΔALB was defined as endogenous ΔALB, reflecting spontaneous albumin change without direct albumin replacement. In patients who received HAS during this period, ΔALB was defined as HAS-exposed ΔALB and was considered potentially influenced by exogenous albumin administration. In MIMIC-IV, HAS exposure during the first 3 ICU days was defined as at least one HAS infusion with a stop time within 72 h after ICU admission.

The primary outcome was 90-day survival. Secondary outcomes were in-hospital survival and overall survival. Follow-up began on the day of admission and continued until death, censoring, or the MIMIC-IV database update (June 23, 2022). Covariates included in the multivariable models are summarized in the Statistical analysis section and listed in detail in Supplementary Appendix 1.

### Statistical analysis

Continuous variables are presented as medians with interquartile ranges (IQRs), and categorical variables are presented as counts and percentages. Groups were compared with the t test or Wilcoxon rank-sum test for continuous variables and with the χ^2^ test or Fisher exact test for categorical variables, as appropriate.

The primary prognostic analyses used multivariable Cox regression models. Restricted cubic splines (RCS) were used to examine potentially nonlinear associations of baseline serum albumin and ΔALB with mortality risk. For RCS analyses, associations were summarized with the global test and the test for nonlinearity. Complete covariate-level outputs are provided in the Supplementary Tables. Covariates in the multivariable Cox models were selected *a priori* on the basis of clinical relevance, prior evidence in sepsis prognosis, plausible biological associations with serum albumin, and data availability in MIMIC-IV. No stepwise variable selection was used for the Cox regression models.

For the baseline serum albumin analysis, Cox models were adjusted for demographic characteristics, chronic comorbidities, early organ support within the first 24 h after ICU admission, ICU day-1 vital signs, and laboratory variables measured during the first 24 h. These variables were treated as fixed early-ICU summary covariates and were not updated during follow-up. Because AKI defined within the first 3 ICU days may represent potentially post-baseline organ dysfunction relative to baseline serum albumin measurement, we repeated the baseline albumin Cox analyses after excluding AKI from the adjustment set.

For the ΔALB–prognosis analyses, patients were stratified according to HAS use during the first 3 ICU days. Multivariable Cox models were fitted separately in the non-HAS and HAS groups. In addition to the prespecified baseline covariates, change in hematocrit (ΔHCT) was included to account for potential hemodilution or hemoconcentration. To address potential temporal mismatch between HAS administration and ΔALB measurement, we performed three time-aligned sensitivity analyses among HAS-treated patients in MIMIC-IV: excluding patients whose ΔALB measurement interval occurred entirely before the first HAS administration, restricting the analysis to patients whose latest albumin measurement occurred after completion of at least one HAS infusion, and performing a strict pre-post HAS analysis including only patients whose first albumin measurement preceded the first HAS administration and whose latest albumin measurement occurred after completion of the first HAS infusion. The same RCS Cox modeling strategy and covariate adjustment as in the primary ΔALB analysis were used.

To assess whether baseline serum albumin modified the association between ΔALB and prognosis, we performed multiplicative interaction analyses in the MIMIC-IV cohort for the primary 90-day outcome. Baseline serum albumin was dichotomized using the 2.77 g/dL threshold identified in the baseline albumin RCS analysis. Cox models were fitted separately in the non-HAS and HAS groups and included ΔALB, baseline albumin category, and the ΔALB × baseline albumin category interaction term, with adjustment for the same covariates as in the primary ΔALB Cox models. *p* values for interaction were obtained using likelihood ratio tests comparing models with and without the interaction term.

To explore factors associated with endogenous ΔALB, we performed multivariable linear regression in the non-HAS cohort, with ΔALB specified as the dependent variable. Candidate variables included baseline demographic and clinical variables, early interventions, vital signs, laboratory variables, changes in vital signs and laboratory variables during the first 3 ICU days, and variables related to fluid balance and nutritional support. Standardized regression coefficients were used to compare the relative strength of associations. Further details regarding covariates and model specifications are provided in Supplementary Appendices 1, 2. The analytical approach for evaluating the population-level effect of HAS administration on serum albumin levels is described in Supplementary Appendix 3.

Missing data were handled using random forest imputation. Data extraction from MIMIC-IV was performed using SQL, and all statistical analyses were conducted using R version 4.2.2 in RStudio. RCS analyses were conducted using the rcssci package in R. All tests were two-sided, and *p* < 0.05 was considered statistically significant.

## Results

### Lower baseline serum albumin was associated with worse prognosis in sepsis

We first examined the relationship between baseline serum albumin and prognosis in patients with sepsis. Baseline serum albumin was defined as the lowest serum albumin concentration measured on ICU day 1. A total of 12,128 patients with available day-1 baseline serum albumin measurements were included. The association was evaluated using restricted cubic splines within prespecified multivariable Cox regression models adjusted for demographic characteristics, chronic comorbidities, early organ support within the first 24 h after ICU admission, ICU day-1 vital signs, and laboratory variables. Baseline characteristics are shown in Supplementary Table S1.

**Figure 2:**
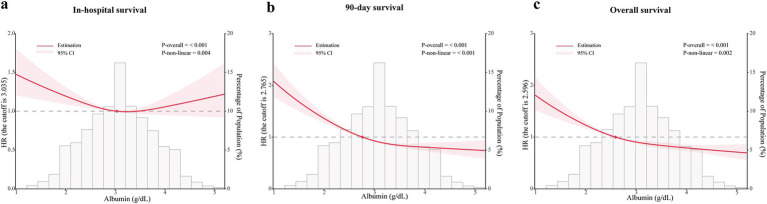
Association of baseline serum albumin with prognosis in septic patients from the MIMIC-IV database (*n* = 12,128). **(a)** In-hospital survival. **(b)** 90-day survival. **(c)** Overall survival. HR, hazard ratio. Models were adjusted for prespecified demographic characteristics, comorbidities, early organ support, ICU day-1 vital signs, and laboratory variables. Complete model outputs are shown in Supplementary Table S2.

As shown in Figures 2a–c, in these adjusted models, baseline serum albumin exhibited significant nonlinear associations with in-hospital, 90-day, and overall outcomes. The RCS global association tests were significant for all three outcomes (all *p* < 0.001), and the tests for nonlinearity were also significant for in-hospital, 90-day, and overall outcomes (*p* = 0.004, *p* < 0.001, and *p* = 0.002, respectively). Lower albumin concentrations were associated with higher mortality risk. For the 90-day outcome, the estimated inflection point was 2.77 g/dL: below this level, decreasing albumin was associated with a progressively higher risk of death, whereas above this threshold the association became less pronounced.

The complete covariate-level outputs of the same adjusted Cox models are provided in Supplementary Table S2. Because AKI defined within the first 3 ICU days could represent potentially post-baseline organ dysfunction relative to baseline serum albumin measurement, we additionally repeated the adjusted RCS Cox analyses after excluding AKI from the adjustment set. The association between baseline serum albumin and all outcomes was materially unchanged in this sensitivity analysis (Supplementary Table S16).

### Endogenous, but not HAS-exposed, ΔALB was associated with prognosis

We next evaluated whether early changes in serum albumin (ΔALB) were associated with prognosis. A total of 3,084 patients met the eligibility criteria for ΔALB assessment during the first 3 ICU days (Supplementary Table S3). In the overall cohort, restricted cubic spline Cox models showed significant overall associations between ΔALB and multiple prognostic outcomes (Supplementary Figure S1). However, evidence for nonlinearity was not consistent across outcomes and was observed mainly for the 90-day outcome. Visual inspection of the adjusted spline curves suggested that higher mortality risk was most apparent among patients with decreases in albumin during the first 3 ICU days. In contrast, positive ΔALB did not show a consistent monotonic association with lower mortality risk in the overall cohort. Because this observation was based on the shape of the spline curves rather than a formal segmented analysis above and below a data-derived cutoff, it should be interpreted descriptively.

**Figure 3:**
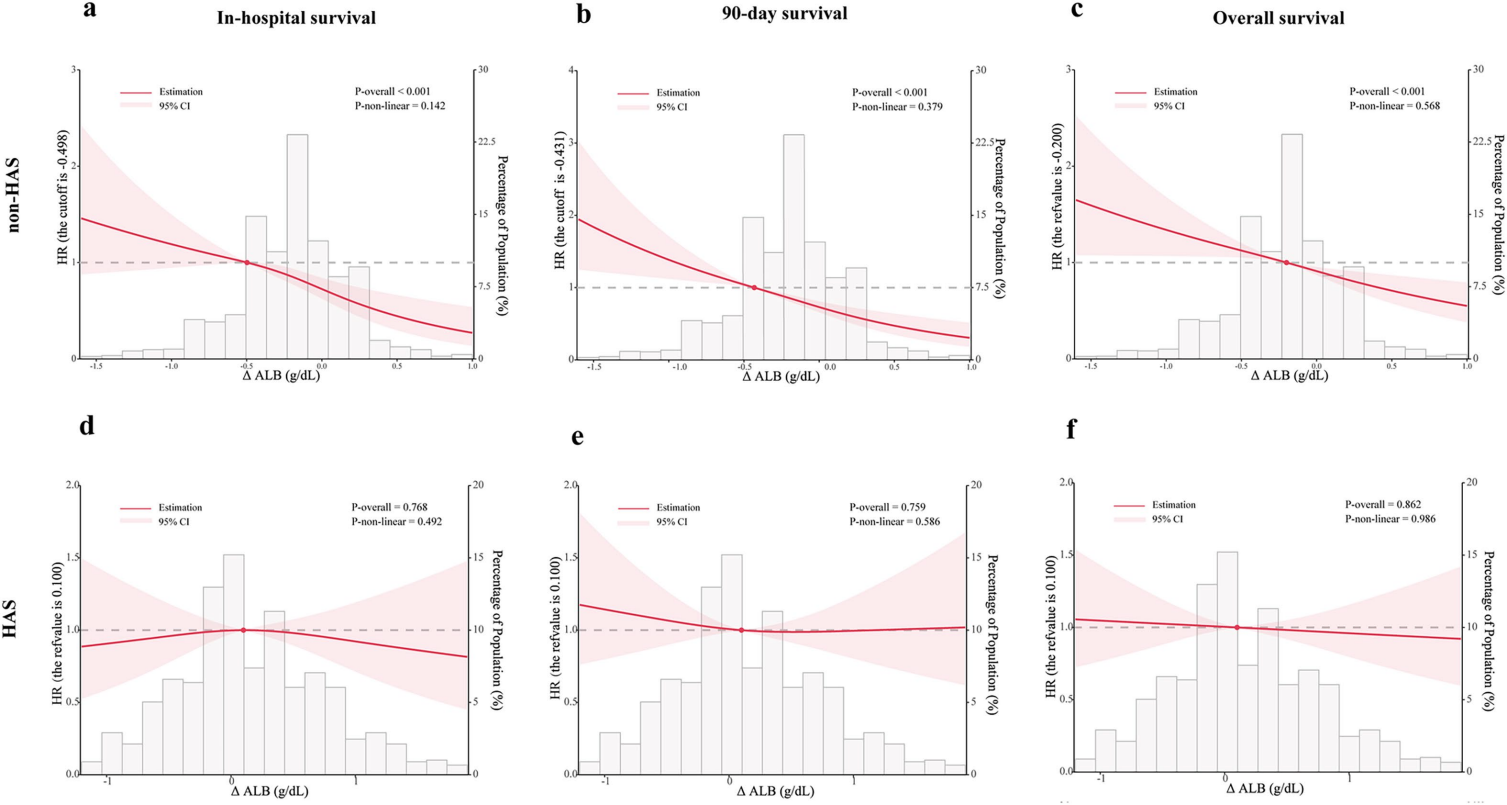
Association of serum albumin change (ΔALB) during the first 3 days after ICU admission with prognostic outcomes in septic patients, stratified by human albumin solution (HAS) use, in the MIMIC-IV database. **(a)** Non-HAS cohort, in-hospital survival. **(b)** Non-HAS cohort, 90-day survival. **(c)** Non-HAS cohort, overall survival. **(d)** HAS cohort, in-hospital survival. **(e)** HAS cohort, 90-day survival. **(f)** HAS cohort, overall survival. HR, hazard ratio; refvalue, reference value used for spline visualization.

To account for the potential influence of exogenous albumin, patients were stratified according to HAS use during the first 3 ICU days into a HAS group (*n* = 911) and a non-HAS group (*n* = 2,173). In the non-HAS group, ΔALB showed a significant association with prognosis, with an approximately linear inverse pattern (Figures 3a–c). Patients in the HAS group received a mean total HAS dose of 83.7 g (95% CI, 78.7–88.6 g). In contrast to the non-HAS group, ΔALB in the HAS group—referred to here as HAS-exposed ΔALB—was not associated with any of the prognostic outcomes examined (Figures 3d–f).

We further examined the timing of serum albumin measurements relative to HAS administration among the 911 HAS-treated patients with calculable ΔALB in MIMIC-IV. In the primary analysis, HAS exposure was defined according to HAS infusion during the first 72 h after ICU admission, and the latest albumin measurement used to calculate ΔALB was not required *a priori* to occur after HAS administration. Among these patients, 37 patients (4.1%) had a ΔALB measurement interval that occurred entirely before the first HAS administration. After excluding these patients, ΔALB remained not significantly associated with 90-day outcome in the restricted cubic spline Cox model (*n* = 874; P-overall = 0.880; P-nonlinearity = 0.751). Similar results were observed when the analysis was restricted to patients whose latest albumin measurement occurred after completion of at least one HAS infusion (*n* = 752; P-overall = 0.921; P-nonlinearity = 0.831). In the strict pre-post HAS cohort, in which the first albumin measurement preceded the first HAS administration and the latest albumin measurement occurred after completion of the first HAS infusion, no significant overall or nonlinear association was observed (*n* = 470; P-overall = 0.279; P-nonlinearity = 0.166). These findings suggest that the lack of prognostic association between HAS-exposed ΔALB and outcomes was not driven by patients whose albumin measurements occurred before HAS exposure (Supplementary Figure S6; Supplementary Tables S15).

Because baseline albumin might modify the prognostic association of ΔALB, we further stratified patients according to whether initial albumin was below or above the previously identified threshold of 2.77 g/dL and performed formal multiplicative interaction analyses (Supplementary Figure S2 and Supplementary Table S17). In the non-HAS group, higher ΔALB was associated with lower 90-day mortality risk in both the low baseline albumin stratum (<2.77 g/dL; HR per 1-g/dL increase = 0.51, 95% CI 0.37–0.71, *p* < 0.001) and the higher baseline albumin stratum (≥2.77 g/dL; HR per 1-g/dL increase = 0.59, 95% CI 0.44–0.80, p < 0.001). There was no significant interaction between baseline albumin category and ΔALB in the non-HAS group (P-interaction = 0.29). In the HAS group, ΔALB was not associated with 90-day mortality in either the low baseline albumin stratum (<2.77 g/dL; HR = 1.08, 95% CI 0.85–1.37, *p* = 0.545) or the higher baseline albumin stratum (≥2.77 g/dL; HR = 1.00, 95% CI 0.76–1.32, *p* = 0.985), and again no significant interaction was observed (P-interaction = 0.67). These findings suggest that baseline albumin level did not materially modify the prognostic association of ΔALB; rather, the prognostic meaning of ΔALB depended primarily on whether the albumin change occurred spontaneously or after HAS exposure.

Taken together, these findings suggest that the prognostic information conveyed by ΔALB in sepsis is driven primarily by endogenous rather than HAS-exposed albumin change, which has implications for interpreting albumin in nutritional assessment.

### Factors associated with endogenous ΔALB in sepsis

**Figure 4:**
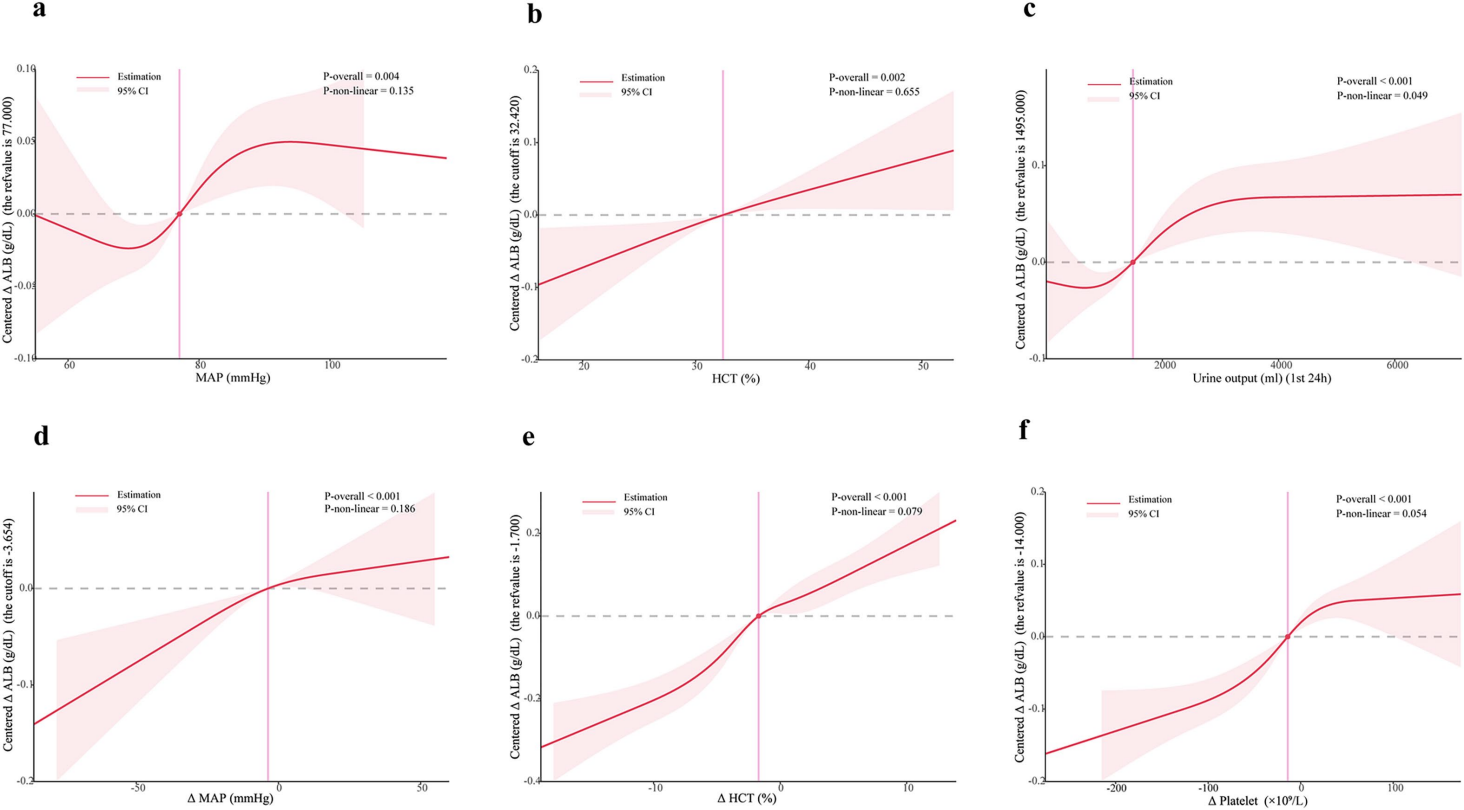
Covariates independently and positively associated with change in serum albumin (ΔALB) during the first 3 days after ICU admission in septic patients, identified by multivariable linear regression. **(a)** Mean arterial pressure (MAP) on ICU day 1. **(b)** Baseline hematocrit (HCT) at ICU admission. **(c)** Total urine output on ICU day 1. **(d)** Change in mean arterial pressure (ΔMAP) during the first 3 days. **(e)** Change in hematocrit (ΔHCT) during the first 3 days. **(f)** Change in platelet count (ΔPLT) during the first 3 days. Cutoff, cutoff value determined using the regression discontinuity design (RDD) method; refvalue, reference point defined by the median of the x-axis parameter.

Given the absence of a prognostic association for HAS-exposed ΔALB, we next focused on factors associated with endogenous ΔALB in the non-HAS cohort (*n* = 2,173). As shown in Supplementary Tables S5, S6, multivariable linear regression demonstrated that mean arterial pressure (MAP), baseline hematocrit, renal replacement therapy, urine output on ICU day 1, and changes in MAP (ΔMAP), hematocrit (ΔHCT), and platelet count (ΔPLT) during the first 3 days were positively associated with ΔALB (Figures 4a–f).

**Figure 5:**
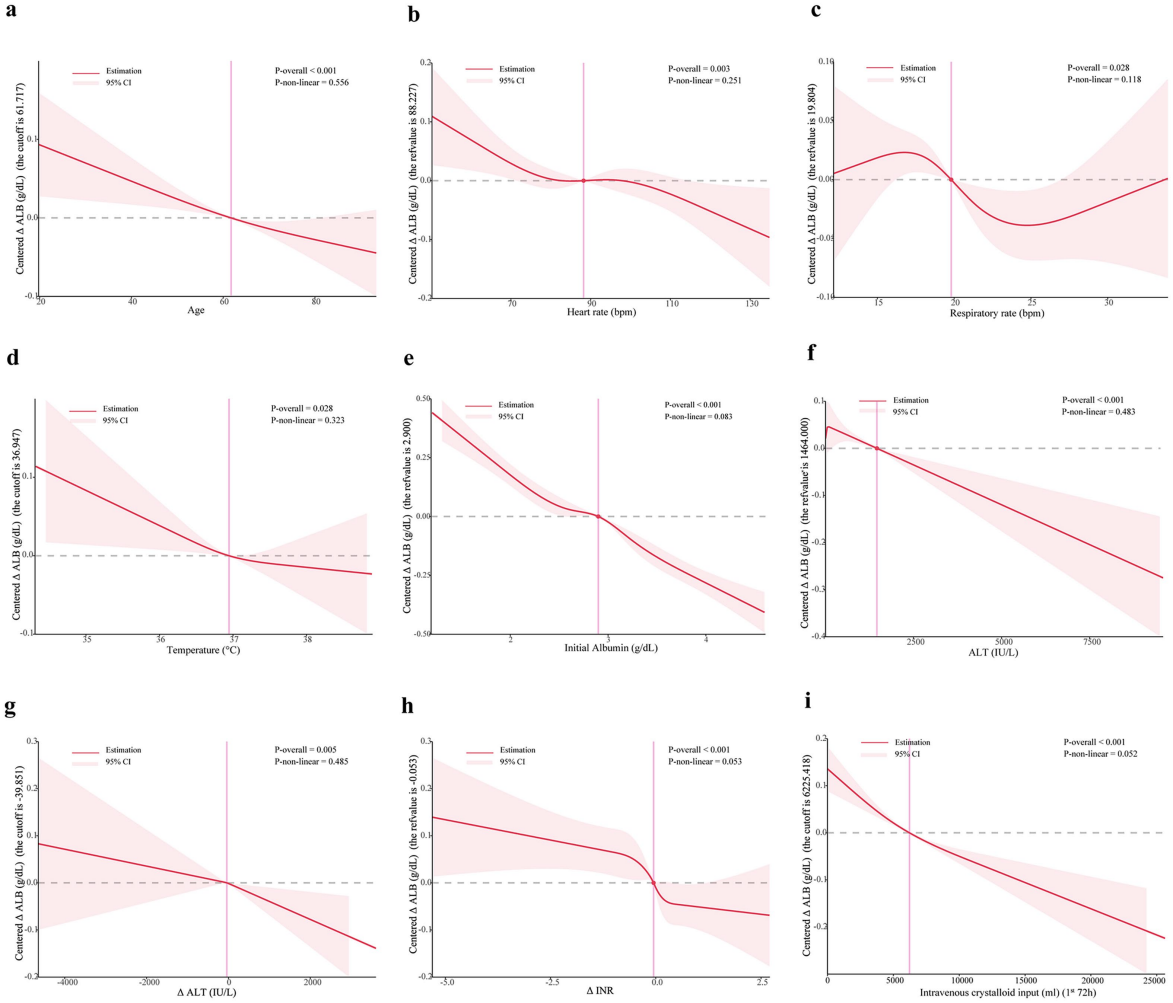
Covariates independently and negatively associated with change in serum albumin (ΔALB) during the first 3 days after ICU admission in septic patients, identified by multivariable linear regression. **(a)** Age at ICU admission. **(b)** Mean heart rate on ICU day 1. **(c)** Mean respiratory rate on ICU day 1. **(d)** Mean body temperature on ICU day 1. **(e)** Baseline serum albumin level (lowest albumin on day 1) at ICU admission. **(f)** Peak alanine aminotransferase (ALT) on ICU day 1. **(g)** Change in ALT during the first 3 days. **(h)** Change in international normalized ratio (INR) during the first 3 days. **(i)** Total intravenous crystalloid volume during the first 3 days after ICU admission. HR, hazard ratio; refvalue, reference value used for spline visualization.

By contrast, age, mean heart rate, mean body temperature, mean respiratory rate, peak alanine aminotransferase (ALT), baseline serum albumin on ICU day 1, change in ALT (ΔALT), change in international normalized ratio (ΔINR), and intravenous crystalloid volume during the first 3 days were negatively associated with ΔALB (Figures 5a–i).

To compare the relative contributions of these variables, we examined standardized regression coefficients (*β*). The strongest associations were observed for baseline serum albumin on day 1 (β = −0.359), ΔHCT (β = 0.254), intravenous crystalloid volume during the first 3 days (β = −0.176), ΔPLT (β = 0.122), and peak ALT on day 1 (β = −0.103).

### External cohort analysis of ΔALB–prognosis associations

We further examined ΔALB–prognosis associations in an independent external cohort from the First Affiliated Hospital of Xi’an Jiaotong University. Among this cohort, 689 patients met the eligibility criteria for ΔALB analysis. Baseline characteristics are shown in Supplementary Table S4. Of these patients, 670 patients (97.2%) received HAS during the first 3 ICU days, leaving only 19 HAS-free patients. Therefore, this cohort was not suitable for formal external validation of the prognostic association of endogenous ΔALB. Instead, it mainly allowed us to assess whether the findings among HAS-treated patients were directionally consistent in an independent clinical setting. In the overall cohort and in the HAS subgroup, ΔALB showed no clear prognostic association, consistent with the null association observed for HAS-exposed ΔALB in MIMIC-IV (Supplementary Figures S3a,b).

### Effect of HAS on serum albumin levels

Although ΔALB among HAS recipients was not associated with clinical outcomes, albumin infusion remains common in practice. We therefore evaluated its association with short-term albumin change from a broader clinical perspective.

Across these supplementary analyses, HAS administration was associated with an early increase in serum albumin, most consistently within 24 h after infusion. However, the magnitude and persistence of this increase varied according to the analytical unit, HCT correction, completeness of serial measurements, cohort, and time point. In per-infusion analyses, the 72-h change was small and less consistent: uncorrected albumin values were attenuated, unchanged, or decreased in some comparisons, whereas HCT-corrected values showed small increases in several analyses. In per-patient analyses, which captured cumulative HAS exposure during hospitalization, 72-h albumin levels were significantly higher than pre-infusion levels. In multivariable analyses, the total amount of albumin administered remained positively associated with ΔALB during the first 3 ICU days. These findings indicate that HAS administration was associated with increases in measured serum albumin, particularly early after infusion, but the persistence of this effect was heterogeneous and HAS-exposed albumin changes were not associated with prognosis (Supplementary Figures S4, S5 and Supplementary Tables S7–S14).

## Discussion

In this real-world study, the primary MIMIC-IV analyses suggested that the prognostic significance of early serum albumin change in sepsis depended strongly on albumin source. In patients not exposed to HAS, spontaneous increases in serum albumin were associated with improved outcomes, whereas HAS-exposed albumin changes were not. The external cohort, in which nearly all patients received HAS, mainly provided a consistency assessment for the absence of a clear prognostic association among HAS-treated patients rather than a validation of endogenous ΔALB. Importantly, endogenous ΔALB in MIMIC-IV was also associated with variables related to hemodynamics, fluid balance, platelet dynamics, and liver function. These findings suggest that early albumin recovery in sepsis is not simply a numerical laboratory change, but a context-dependent biomarker whose interpretation extends beyond nutritional status alone.

This distinction may help explain the long-standing discrepancy between the prognostic value of hypoalbuminemia and the limited survival benefit observed with albumin infusion in randomized trials. In sepsis, serum albumin is shaped by multiple non-nutritional and illness-related processes, including endothelial barrier disruption, capillary leakage, redistribution of intravascular fluid, altered hepatic synthesis, oxidative stress, and treatment-related hemodilution (14–21). Under these conditions, spontaneous recovery of albumin may reflect broader physiological stabilization rather than nutritional repletion per se. By contrast, exogenous albumin infusion may increase measured albumin levels without substantially modifying the underlying pathophysiology that drives prognosis. This interpretation is consistent with major randomized studies, including SAFE (22) and ALBIOS (9), which did not demonstrate a clear survival benefit of albumin therapy in sepsis.

The source-dependent nature of albumin change is therefore central to nutritional interpretation. Because serum albumin is often regarded as a nutrition-related serum protein, clinicians may be tempted to interpret a rising albumin level as evidence of nutritional improvement. Our data do not support that assumption in sepsis. Within the MIMIC-IV cohort, by separating patients according to HAS exposure during the first 3 ICU days, we were able to examine albumin dynamics in two biologically distinct contexts: spontaneous endogenous recovery and exogenous change potentially influenced by replacement therapy. In MIMIC-IV, only the former was associated with improved outcomes. Formal interaction analyses did not show clear evidence that baseline albumin category materially modified the association between ΔALB and 90-day prognosis in either the non-HAS or HAS group, suggesting that the source of albumin change, rather than the initial albumin level alone, was the more important determinant of its prognostic interpretation. Notably, this pattern remained consistent across strata defined by baseline serum albumin level, including patients with marked hypoalbuminemia before infusion. These findings do not support the assumption that increasing serum albumin concentration per se represents nutritional recovery or improves prognosis in sepsis. Importantly, this interpretation was supported by time-aligned sensitivity analyses in MIMIC-IV. Only 4.1% of HAS-treated patients had ΔALB calculated entirely before the first HAS administration, and exclusion of these patients, as well as restriction to patients with post-infusion albumin measurements, did not materially change the null association between HAS-exposed ΔALB and prognosis.

The factors associated with endogenous ΔALB further support the view that albumin dynamics reflect broader physiological processes relevant to critical illness. The inverse association with crystalloid volume and the positive association with ΔHCT suggest that hemodilution and hemoconcentration contribute importantly to early albumin changes. Associations with platelet change and vital signs may reflect illness severity, inflammatory burden, endothelial dysfunction, and circulatory status. The negative associations with ALT and INR are also consistent with impaired hepatic function contributing to reduced endogenous albumin recovery. Taken together, these findings suggest that serum albumin change in sepsis should not be interpreted in isolation, but rather within a broader framework of fluid status, organ dysfunction, treatment exposure, and disease severity. Further details regarding these associations are provided in Supplementary Appendix 5.

From a clinical nutrition perspective, our findings argue against using serum albumin alone as a surrogate marker of nutritional state or response to nutritional support in septic ICU patients. This interpretation is consistent with the ASPEN position paper, which emphasizes that visceral proteins such as serum albumin primarily reflect inflammation, illness severity, and nutrition risk rather than protein-energy malnutrition or total body protein stores (23). Instead, albumin should be understood as a nutrition-related but nonspecific biomarker that is strongly modified by inflammation, vascular leakage, fluid resuscitation, and hepatic function. Nutritional assessment in critically ill patients should therefore remain multidimensional and, when possible, integrate clinical history, weight loss, muscle mass or body composition, inflammatory burden, fluid balance, hepatic function, and actual nutrition support delivery rather than relying on serum albumin concentration alone. This view aligns with the broader principle that management of sepsis should prioritize control of infection, stabilization of hemodynamics, and treatment of organ dysfunction rather than correction of laboratory abnormalities in isolation (4, 24).

Our study has limitations. First, its retrospective design precludes causal inference and leaves room for residual confounding. Although we adjusted for multiple early severity, organ dysfunction, hemodynamic, hematologic, hepatic, renal, and fluid-related variables, detailed data on some aspects of sepsis management, such as infection source, antimicrobial timing, source control, vasopressor dose and duration, and the clinical indication for HAS administration, were unavailable or could not be reliably standardized across datasets. Thus, the findings should be interpreted as prognostic and context-informative rather than causal. Second, variation in the timing and frequency of laboratory measurements may have affected estimation of ΔALB. Because ΔALB required at least two serum albumin measurements during the first 72 h, the ΔALB-related findings apply primarily to patients with observable early albumin dynamics. Third, nutritional assessment variables, such as pre-ICU nutritional status, muscle mass, weight loss, and caloric or protein adequacy, were unavailable; thus, our nutritional interpretation concerns the clinical use of serum albumin as a nutrition-related biomarker rather than direct diagnosis of malnutrition. Fourth, because 670 of 689 patients in the external cohort received HAS, the external cohort did not provide a sufficiently sized HAS-free subgroup for robust validation of the prognostic significance of endogenous ΔALB. Therefore, the external cohort mainly assessed the consistency of findings among HAS-treated patients. Prospective studies are needed to confirm these observations and to better define the biological mechanisms underlying albumin dynamics in sepsis.

## Conclusion

In patients with sepsis, lower baseline serum albumin was associated with worse outcomes. In the primary MIMIC-IV cohort, spontaneous increases in serum albumin during the first 3 ICU days were associated with better prognosis, whereas HAS-exposed albumin changes were not; the external cohort mainly supported the absence of a clear prognostic association among HAS-treated patients. Endogenous ΔALB appeared to reflect the combined influences of baseline albumin status, hemodilution, illness severity, resuscitation intensity, intravascular concentration or dilution, and liver injury. These findings suggest that in sepsis, serum albumin is better interpreted as a context-dependent, nutrition-related biomarker than as a direct surrogate of nutritional recovery or an isolated therapeutic target for correction.

## Data Availability

The raw data supporting the conclusions of this article will be made available by the authors, without undue reservation.

## Glossary

HAS: Human Albumin Solution
ΔALB: Change in Serum Albumin During the First 3 Days After ICU Admission
ICU: Intensive Care Unit
HR: Hazard Ratio
CI: Confidence Interval
MIMIC-IV: Medical Information Mart for Intensive Care, version IV
SOFA: Sequential Organ Failure Assessment
ΔMAP: Change in Mean Arterial Pressure During the First 3 Days After ICU Admission
ΔHCT: Change in Hematocrit During the First 3 Days After ICU Admission
ΔPLT: Change in Platelet Count During the First 3 Days After ICU Admission
ALT: Alanine Aminotransferase
ΔALT: Change in Alanine Aminotransferase During the First 3 Days After ICU Admission
INR: International Normalized Ratio
ΔINR: Change in International Normalized Ratio During the First 3 Days After ICU Admission
MAP: Mean Arterial Pressure
HCT: Hematocrit
PLT: Platelet Count
RDD: Regression Discontinuity Design
AKI: Acute Kidney Injury
IQR: Interquartile Range
RRT: Renal Replacement Therapy
SAFE: Saline versus Albumin Fluid Evaluation Trial
ALBIOS: Albumin Italian Outcome Sepsis Trial

## References

[1] Gotts JE Matthay MA. Sepsis: pathophysiology and clinical management. BMJ. (2016) 353:i1585. doi: 10.1136/bmj.i1585, 27217054

[2] Vincent JL Jones G David S Olariu E Cadwell KK. Frequency and mortality of septic shock in Europe and North America: a systematic review and meta-analysis. Crit Care. (2019) 23:196. doi: 10.1186/s13054-019-2478-6, 31151462 PMC6545004

[3] Liu V Escobar GJ Greene JD Soule J Whippy A Angus DC . Hospital deaths in patients with sepsis from 2 independent cohorts. JAMA. (2014) 312:90–2. doi: 10.1001/jama.2014.5804, 24838355

[4] Cecconi M Evans L Levy M Rhodes A. Sepsis and septic shock. Lancet. (2018) 392:75–87. doi: 10.1016/S0140-6736(18)30696-2 29937192

[5] Reinhart K Daniels R Kissoon N Machado FR Schachter RD Finfer S. Recognizing Sepsis as a Global Health priority - a WHO resolution. N Engl J Med. (2017) 377:414–7. doi: 10.1056/NEJMp1707170, 28658587

[6] Wiedermann CJ. Hypoalbuminemia as surrogate and culprit of infections. Int J Mol Sci. (2021) 22:22. doi: 10.3390/ijms22094496, 33925831 PMC8123513

[7] Caironi P. POINT: should intravenous albumin be used for volume resuscitation in severe sepsis/septic shock? Yes. Chest. (2016) 149:1365–7. doi: 10.1016/j.chest.2016.03.048, 27287566

[8] Coz YA Flannery AH Simpson SQ. COUNTERPOINT: should intravenous albumin be used for volume resuscitation in severe sepsis/septic shock? No. Chest. (2016) 149:1368–70. doi: 10.1016/j.chest.2016.03.050 27287567

[9] Caironi P Tognoni G Masson S Fumagalli R Pesenti A Romero M . Albumin replacement in patients with severe Sepsis or septic shock. N Engl J Med. (2014) 370:1412–21. doi: 10.1056/NEJMoa1305727, 24635772

[10] Callum J Skubas NJ Bathla A Keshavarz H Clark EG Rochwerg B . Use of intravenous albumin: a guideline from the international collaboration for transfusion medicine guidelines. Chest. (2024) 166:321–38. doi: 10.1016/j.chest.2024.02.049, 38447639 PMC11317816

[11] Humpert PM Lukic IK Thorpe SR Hofer S Awad EM Andrassy M . AGE-modified albumin containing infusion solutions boosts septicaemia and inflammation in experimental peritonitis. J Leukoc Biol. (2009) 86:589–97. doi: 10.1189/jlb.1008646, 19401390 PMC2735283

[12] Tigabu BM Davari M Kebriaeezadeh A Mojtahedzadeh M Sadeghi K Najmeddin F . A cost-effectiveness analysis of albumin in septic shock: a patient-level data analysis. Clin Ther. (2019) 41:2297–2307.e2. doi: 10.1016/j.clinthera.2019.08.023, 31668842

[13] Johnson A Bulgarelli L Pollard T Horng S Celi LA Mark R. MIMIC-IV (version 2.0). PhysioNet. (2022). doi: 10.13026/7vcr-e114.

[14] Patel A Laffan MA Waheed U Brett SJ. Randomised trials of human albumin for adults with sepsis: systematic review and meta-analysis with trial sequential analysis of all-cause mortality. BMJ. (2014) 349:g4561. doi: 10.1136/bmj.g4561, 25099709 PMC4106199

[15] Vincent JL Russell JA Jacob M Martin G Guidet B Wernerman J . Albumin administration in the acutely ill: what is new and where next? Crit Care. (2014) 18:231. doi: 10.1186/cc13991, 25042164 PMC4223404

[16] Erstad BL Gales BJ Rappaport WD. The use of albumin in clinical practice. Arch Intern Med. (1991) 151:901–11. 1902657

[17] Oettl K Birner-Gruenberger R Spindelboeck W Stueger HP Dorn L Stadlbauer V . Oxidative albumin damage in chronic liver failure: relation to albumin binding capacity, liver dysfunction and survival. J Hepatol. (2013) 59:978–83. doi: 10.1016/j.jhep.2013.06.013, 23811308

[18] Giannone FA Domenicali M Baldassarre M Bartoletti M Naldi M Laggetta M . Ischaemia-modified albumin: a marker of bacterial infection in hospitalized patients with cirrhosis. Liver Int. (2015) 35:2425–32. doi: 10.1111/liv.12860, 25939693

[19] O'Brien AJ Fullerton JN Massey KA Auld G Sewell G James S . Immunosuppression in acutely decompensated cirrhosis is mediated by prostaglandin E2. Nat Med. (2014) 20:518–23. doi: 10.1038/nm.3516, 24728410 PMC5369639

[20] Zhang J Li X He R Zheng W Kwong JS Lu L . The effectiveness of clinical pharmacist-led consultation in the treatment of infectious diseases: a prospective, multicenter, cohort study. Front Pharmacol. (2020) 11:575022. doi: 10.3389/fphar.2020.575022, 33013418 PMC7506045

[21] Ferrer R Mateu X Maseda E Yebenes JC Aldecoa C De Haro C . Non-oncotic properties of albumin. A multidisciplinary vision about the implications for critically ill patients. Expert Rev Clin Pharmacol. (2018) 11:125–37. doi: 10.1080/17512433.2018.1412827, 29219627

[22] Finfer S Bellomo R Boyce N French J Myburgh J Norton R. A comparison of albumin and saline for fluid resuscitation in the intensive care unit. N Engl J Med. (2004) 350:2247–56. doi: 10.1056/NEJMoa040232 15163774

[23] Evans DC Corkins MR Malone A Miller S Mogensen KM Guenter P . The use of visceral proteins as nutrition markers: an ASPEN position paper. Nutr Clinic Pract. (2021) 36:22–8. doi: 10.1002/ncp.10588, 33125793

[24] Vincent JL. Current sepsis therapeutics. EBioMedicine. (2022) 86:104318. doi: 10.1016/j.ebiom.2022.104318, 36470828 PMC9782815

