# Supplementary material for: Early serum albumin dynamics in sepsis differ by albumin source and have implications for nutritional assessment
Source: Front Nutr. 2026 Jul 9;13:1858939. doi: 10.3389/fnut.2026.1858939 (PMC13391287; doi:10.3389/fnut.2026.1858939)
Supplement: Supplementary file 1 [file Table_1.docx]

**Early serum albumin dynamics in sepsis differ by albumin source and have implications for nutritional assessment**

Running title: Albumin dynamics and nutritional assessment in sepsis

Yifei Ma, M.D.^1,5,6^, Kongyuan Wei, M.D.^2,6^, Weiqi Lyu, M.D.^1,6^, Yuxuan Tang, M.E.^3^, Tianyi Zhang, M.E.^5^, Qingyong Ma, Ph.D.^1,6^, Li Wen, Ph.D.^4^, Zheng Wang, Ph.D.^1,6^

^1^ Department of Hepatobiliary Surgery, The First Affiliated Hospital of Xi'an Jiaotong University, 277 West Yanta Road, Xi'an, 710061, People's Republic of China

^2^ Department of General, Visceral and Transplantation Surgery, University of Heidelberg, Im Neuenheimer Feld 420, Heidelberg, 69120, Germany

^3^ Huawei Technologies Co., Ltd., 127 Jinye Road, Xi'an, 710100, People's Republic of China

^4^ Peking Union Medical College Hospital, Chinese Academy of Medical Sciences and Peking Union Medical College, Beijing, 100006, People's Republic of China

^5^ Biobank, The First Affiliated Hospital of Xi'an Jiaotong University, 277 West Yanta Road, Xi'an, 710061, People's Republic of China

^6^ Pancreatic Diseases Center of Xi'an Jiaotong University, 277 West Yanta Road, Xi'an, 710061, People's Republic of China

**Author Details**

First author: Yifei Ma, Resident Doctor in Hepatobiliary Surgery, The First Affiliated Hospital of Xi'an Jiaotong University, Email: mayiphy4628@stu.xjtu.edu.cn, ORCID: 0000-0001-5301-5562, Address: 277 Yanta Western Rd., Xi'an, 710061, Shaanxi Province, People's Republic of China,

Co-first author: Kongyuan Wei, M.D., Resident Doctor in Hepatobiliary Surgery, The First Affiliated Hospital of Xi'an Jiaotong University, Email: jasonwky@163.com, Address: 277 Yanta Western Rd., Xi'an, 710061, Shaanxi Province, People's Republic of China,

Corresponding author: Zheng Wang, Prof., Ph.D., Professor in Hepatobiliary Surgery, The First Affiliated Hospital of Xi'an Jiaotong University, Tel: (+86) 029-85324695, Email: zheng.wang11@mail.xjtu.edu.cn, ORCID: 0000-0002-0490-466X, Address: 277 Yanta Western Rd., Xi'an, 710061, Shaanxi Province, People's Republic of China.

### Supplementary Appendix 1: Variables Included in the Multivariable Analyses:

Covariates were selected on the basis of clinical relevance, prior evidence in sepsis prognosis, plausible biological associations with serum albumin, and data availability in MIMIC-IV. Because serum albumin is affected by inflammation, capillary leak, fluid redistribution, hepatic function, renal function, and intravascular dilution or concentration, the models included available variables representing these domains.

1.Covariates for baseline serum albumin–prognosis Cox models

For Cox models evaluating the association between baseline serum albumin and prognosis, baseline serum albumin was defined as the lowest serum albumin concentration measured on ICU day 1 and was modeled using restricted cubic splines. The adjustment set included age, sex, body weight at admission, congestive heart failure, chronic obstructive pulmonary disease, diabetes, malignancy, renal replacement therapy within the first 24 hours, mechanical ventilation within the first 24 hours, AKI, mean heart rate on ICU day 1, mean arterial pressure on ICU day 1, mean respiratory rate on ICU day 1, mean body temperature on ICU day 1, lowest platelet count during the first 24 hours, highest white blood cell count during the first 24 hours, baseline hematocrit, highest alanine aminotransferase during the first 24 hours, highest serum creatinine during the first 24 hours, and highest lactate during the first 24 hours. Dynamic 72-hour change variables were not included in the baseline serum albumin Cox models. Because AKI defined within the first 3 ICU days may occur after baseline albumin measurement, sensitivity analyses were performed after excluding AKI from the adjustment set.

2.Covariates for ΔALB–prognosis Cox models

For Cox models evaluating the association between ΔALB and prognosis, ΔALB was modeled using restricted cubic splines, and analyses were stratified by HAS exposure during the first 3 ICU days. The adjustment set included age, sex, body weight at admission, congestive heart failure, chronic obstructive pulmonary disease, diabetes, malignancy, renal replacement therapy within the first 24 hours, mechanical ventilation within the first 24 hours, AKI, mean heart rate on ICU day 1, mean arterial pressure on ICU day 1, mean respiratory rate on ICU day 1, mean body temperature on ICU day 1, lowest platelet count during the first 24 hours, highest white blood cell count during the first 24 hours, baseline hematocrit, change in hematocrit during the first 3 ICU days, baseline serum albumin, highest alanine aminotransferase during the first 24 hours, highest serum creatinine during the first 24 hours, and highest lactate during the first 24 hours.

3.Covariates for baseline albumin × ΔALB interaction analyses

For the interaction analyses, baseline serum albumin was dichotomized as <2.77 g/dL versus ≥2.77 g/dL using the threshold identified in the baseline albumin RCS analysis. Cox models were fitted separately in the non-HAS and HAS groups and included ΔALB, baseline albumin category, and the ΔALB × baseline albumin category interaction term. The adjustment set was the same as that used in the primary ΔALB Cox models, except that baseline serum albumin was represented by the dichotomized baseline albumin category.

4.Covariates for analyses of factors associated with endogenous ΔALB

To identify factors associated with endogenous ΔALB, analyses were restricted to patients without HAS exposure during the first 3 ICU days. Candidate variables included age, sex, body weight at admission, congestive heart failure, chronic obstructive pulmonary disease, diabetes, malignancy, renal replacement therapy within the first 24 hours, mechanical ventilation within the first 24 hours, AKI, mean heart rate, mean arterial pressure, mean respiratory rate, mean body temperature, platelet count, white blood cell count, hematocrit, alanine aminotransferase, serum creatinine, blood lactate, blood glucose, international normalized ratio, baseline serum albumin, changes in vital signs during the first 3 ICU days (Δheart rate, ΔMAP, Δrespiratory rate, and Δbody temperature), changes in laboratory variables during the first 3 ICU days (Δplatelet count, ΔWBC, ΔHCT, ΔALT, Δcreatinine, Δblood glucose, and ΔINR), urine output during the first 24 hours, total intravenous crystalloid volume during the first 3 ICU days, and enteral nutrition volume during the first 3 ICU days.

5.Covariates for HAS dose–ΔALB analyses

In analyses examining the association between administered HAS dose and ΔALB among HAS-treated patients, the total HAS dose administered during the first 3 ICU days was the primary independent variable. Models were adjusted using covariates similar to those used in the endogenous ΔALB determinant analysis, with additional consideration of variables related to fluid status and hemodilution.

### Supplementary Appendix 2: Statistical Analysis Methods:

Continuous variables are presented as medians with interquartile ranges (IQRs). Categorical variables are presented as counts and percentages. Continuous variables were compared using the t test or Wilcoxon rank-sum test, as appropriate, and categorical variables were compared using the χ² test or Fisher exact test.

Survival outcomes included in-hospital survival, 90-day survival, and overall survival. The primary outcome was 90-day survival. Multivariable Cox regression models were used to evaluate associations between serum albumin variables and mortality risk. Restricted cubic splines (RCS) were used to allow for potentially nonlinear associations of baseline serum albumin and ΔALB with outcomes. For RCS analyses, P-overall represents the global association test, and P-nonlinearity represents the test for departure from linearity. Because baseline serum albumin and ΔALB were modeled using RCS in the primary analyses, these associations are primarily summarized using global and nonlinearity tests rather than a single linear hazard ratio. Complete covariate-level Cox model outputs, including hazard ratios, 95% confidence intervals, and P values for model covariates, are provided in the supplementary tables.

For analyses of baseline serum albumin, the lowest serum albumin concentration measured on ICU day 1 was treated as the primary independent variable. For analyses of early albumin dynamics, ΔALB was defined as the latest serum albumin value minus the earliest serum albumin value during the first 72 hours after ICU admission, with a minimum interval of 24 hours between the two measurements. ΔALB analyses were stratified according to HAS exposure during the first 3 ICU days.

For HAS-treated patients with calculable ΔALB in MIMIC-IV, the timing of albumin measurements relative to HAS administration was evaluated. HAS administration was identified using infusion start and stop times, and HAS exposure during the first 3 ICU days was defined as at least one HAS infusion with stop time within 72 hours after ICU admission. Three nested time-aligned sensitivity analyses were performed: (1) excluding patients whose ΔALB measurement interval occurred entirely before the first HAS administration; (2) restricting the analysis to patients whose latest albumin measurement occurred after completion of at least one HAS infusion; and (3) restricting the analysis to patients with a strict pre-post HAS pattern, defined as first albumin measurement before first HAS administration and latest albumin measurement after completion of the first HAS infusion. The same RCS Cox modeling strategy and covariate adjustment as in the primary ΔALB analysis were used.

To assess whether baseline serum albumin modified the prognostic association of ΔALB, multiplicative interaction analyses were performed for the primary 90-day outcome in the MIMIC-IV cohort. Baseline serum albumin was dichotomized as <2.77 g/dL versus ≥2.77 g/dL. Cox models with and without the ΔALB × baseline albumin category interaction term were compared using likelihood ratio tests. These analyses were performed separately in the non-HAS and HAS groups.

To identify factors associated with endogenous ΔALB, multivariable linear regression was performed among patients without HAS exposure during the first 3 ICU days, with ΔALB specified as the dependent variable. Candidate variables are listed in Supplementary Appendix 1. Stepwise variable selection was used for this exploratory linear regression analysis. Nonlinear relationships between continuous covariates and ΔALB were assessed using RCS within the regression framework. Standardized regression coefficients (β) were used to compare the relative strength of associations between covariates and ΔALB.

Missing data were handled using random forest imputation to reduce bias and preserve sample size. Data extraction from MIMIC-IV was performed using SQL, and all statistical analyses were conducted using R version 4.2.2 in RStudio. RCS analyses were conducted using the rcssci package in R. All statistical tests were two-sided, and P < 0.05 was considered statistically significant.

### Supplementary Appendix 3: Analytical Approach for Evaluating the Effect of HAS Administration on Serum Albumin Levels at the Population Level:

This analysis included patients with sepsis who received at least one administration of human albumin solution (HAS) and had at least two serum albumin measurements during hospitalization. Based on these criteria, we identified 20,909 HAS infusion records and 31,999 serum albumin measurements from 3,946 patients. In the MIMIC-IV database, each HAS administration for an individual patient (identified by a unique pharmacy_id) is recorded with a start time and stop time, indicating the infusion interval. The administered albumin dose in grams is also recorded. Serum albumin and hematocrit measurements each have their own chart time indicating the time of sampling. In contrast, in the cohort from the First Affiliated Hospital of Xi'an Jiaotong University, HAS records included only the administration start time, whereas laboratory time stamps were recorded similarly to those in MIMIC-IV.

The primary objective of this analysis was to identify an eligible baseline serum albumin value before each HAS administration and to determine whether valid post-infusion serum albumin measurements were available approximately 1, 3, and 7 days after infusion completion. Given an eligible baseline measurement, we analyzed cohorts with at least one available post-infusion measurement at these time points, as well as cohorts with all three post-infusion measurements available. To provide a broader patient-level perspective, we also analyzed all HAS administrations during a single hospitalization as a combined exposure unit.

Initially, each HAS administration episode was treated as a separate infusion unit. The most recent serum albumin measurement obtained within 72 hours before the start of a given infusion was defined as the eligible pre-infusion baseline albumin level for that episode, provided that no other HAS administration occurred between that measurement and the start of the infusion.

Post-infusion albumin measurements were defined as follows:

1.The earliest serum albumin measurement obtained within 0-24 hours after the end of the infusion, provided that no additional HAS was administered in the interim, was defined as the albumin level within 24 hours post-infusion.

2.Under the same condition, the earliest serum albumin measurement obtained within 48-96 hours after infusion completion was defined as the albumin level approximately 3 days post-infusion.

3.Likewise, the earliest serum albumin measurement obtained within 6-14 days after infusion completion was defined as the albumin level approximately 7 days post-infusion.

We also conducted a patient-level analysis in which all HAS administrations during a hospitalization were treated as a single HAS exposure unit. In this analysis, the most recent serum albumin measurement within 72 hours before the first HAS administration during that hospitalization was defined as the baseline albumin level before all HAS administrations. The earliest serum albumin measurements obtained within 0-24 hours, 48-96 hours, and 6-14 days after completion of the final HAS administration were then defined as post-infusion albumin levels at different time points.

In addition, hematocrit values measured over the same time windows were identified using the same criteria, allowing calculation of hematocrit-corrected albumin concentrations to reduce the potential influence of hemodilution. HCT-corrected albumin concentrations were calculated according to the following formula^1^:

$${Alb}_{t(corr)}=Alb_{t}*\frac{1-{100}/{Hct_{t}}}{{1-100}/{Hct_{0}}}$$

where ${Alb}_{t(corr)}$ is the HCT-corrected albumin concentration at time *t*, $Alb_{t}$ is the actual albumin concentration at time *t*, $Hct_{t}$ is the hematocrit at time *t*, and $Hct_{0}$ is the baseline hematocrit level.

**References**

1. Margarson MP, Soni NC, (2004) Changes in serum albumin concentration and volume expanding effects following a bolus of albumin 20% in septic patients. BRIT J ANAESTH 92: 821-826

### Supplementary Appendix 4: Effect of HAS on Serum Albumin Levels in Patients With Sepsis:

Although changes in serum albumin levels among HAS recipients during the first 3 days after ICU admission were not significantly associated with clinical outcomes, albumin administration remains common in clinical practice. We therefore examined changes in serum albumin before and after HAS administration from a pragmatic population-level perspective. A schematic overview of the analytical design is shown in e-Appendix 3.

In the MIMIC-IV cohort, with each HAS administration treated as an individual study unit, 218 infusion episodes had eligible serum albumin measurements before infusion and at 24 hours, 3 days, and 7 days after infusion. The mean administered albumin dose per infusion was 41.6 g (95% CI, 37.8-45.4 g). The first post-infusion albumin measurement, obtained within 24 hours, showed a mean increase of 0.203 g/dL relative to the pre-infusion level. By approximately 3 days after infusion, however, serum albumin levels no longer differed significantly from baseline. At approximately 7 days, serum albumin levels were significantly lower than pre-infusion levels, with a mean decrease of 0.089 g/dL. This pattern was more evident in the cohort with incomplete serial measurements, in which serum albumin decreased by 0.044 g/dL at approximately 3 days after 968 infusions (P=0.007) and by 0.110 g/dL at approximately 7 days after 578 infusions. These results are shown in Figure S4a-b and Tables S7-8.

We also analyzed albumin levels before and after all HAS administrations during a given hospitalization, with each patient treated as the study unit. A total of 137 patients had eligible albumin measurements at all four time points. The mean total albumin dose administered during hospitalization was 208.7 g (95% CI, 159.9-257.5 g), and the mean interval from initiation of the first infusion to completion of the final infusion was 13.1 days (95% CI, 9.7-16.5 days). In this patient-level analysis, serum albumin levels increased by 0.425 g/dL, 0.239 g/dL, and 0.160 g/dL at 24 hours, 3 days, and 7 days, respectively, after completion of all albumin infusions compared with the level before the first infusion; all differences were statistically significant. Among patients with only one pre-infusion and one post-infusion albumin measurement, smaller but still significant increases were also observed (0.379 g/dL, 0.149 g/dL, and 0.091 g/dL, respectively), as shown in Tables S9-10.

Within the HAS group, we performed RCS analyses in multivariable linear regression models using ΔALB as the dependent variable and the total HAS dose administered during the preceding 3 days as the independent variable. Covariates were similar to those used in Result 3 of the main manuscript. These analyses showed a significant positive association between HAS dose and ΔALB. Thus, although HAS-related albumin change was not associated with prognosis, greater albumin exposure remained independently associated with larger short-term increases in serum albumin (Figure S5a-b).

The effect of HAS on serum albumin levels in the external validation cohort was broadly consistent with the MIMIC-IV findings. In that cohort, no significant difference was observed between serum albumin levels before infusion and approximately 3 days after a single mean HAS dose of 20.50 g (95% CI, 20.16-20.84 g; P=0.208). However, infused albumin dose remained positively associated with ΔALB in multivariable analysis. Detailed results are shown in Figure S4c-d, Figure S5b, and Tables S11-14.

These analyses indicate that HAS administration was associated with an early increase in measured serum albumin, particularly within 24 hours after infusion. However, the magnitude and persistence of this increase varied by analytical unit, completeness of serial measurements, HCT correction, cohort, and time point. In per-infusion analyses, the 72-hour effect was generally smaller and less consistent, whereas per-patient analyses, which captured cumulative HAS exposure during hospitalization, more often showed persistent increases at 72 hours. Across analyses, greater HAS dose remained positively associated with short-term albumin change. Therefore, HAS exposure can increase measured serum albumin, but HAS-exposed albumin changes should not be interpreted as equivalent to endogenous physiological recovery or as a prognostic marker.

### Supplementary Appendix 5: Detailed Discussion of Factors Associated With Early Changes in Serum Albumin:

Overall, our findings suggest that a greater inflammatory burden at ICU admission may predict a greater decline, or a smaller recovery, in serum albumin over the subsequent 3 days, consistent with previous reports.[1,2] In addition to the positive association between mean arterial pressure (MAP) on day 1 and ΔALB, mean heart rate, mean respiratory rate, and mean body temperature were all negatively associated with ΔALB. These findings may reflect the severity of systemic inflammatory response and further underscore the importance of timely and adequate resuscitation. We also observed a positive association between urine output on ICU day 1 and ΔALB, particularly when urine volume ranged from 1000 to 3000 mL. In critically ill patients, greater urine output is often considered a marker of early resuscitation success and recovery and may also reflect better renal perfusion. By contrast, greater crystalloid administration was inversely associated with ΔALB. Higher baseline hematocrit and increases in hematocrit over the first 3 days were suggestive of hemoconcentration, whereas renal replacement therapy may indirectly increase ΔALB by removing excess fluid and thereby concentrating circulating albumin.

With respect to liver-related variables, both peak alanine aminotransferase (ALT) on ICU day 1 and change in ALT (ΔALT) over the first 3 days were negatively associated with ΔALB during the same period. As the liver is the principal site of albumin synthesis, these findings suggest that early hepatocellular injury and worsening liver dysfunction may limit endogenous albumin recovery in sepsis. Notably, previous studies have shown that albumin fractional synthetic rate does not necessarily decrease during sepsis and may even increase.[3-5] Because ALT primarily reflects acute hepatocellular injury, our findings support a clinically relevant link between early liver injury and impaired albumin recovery.

We also identified notable associations between coagulation-related variables and albumin dynamics. Change in platelet count (ΔPLT) was positively associated with ΔALB, particularly when ΔPLT was below 70, whereas change in international normalized ratio (ΔINR) was negatively associated with ΔALB. These findings may reflect shared links between inflammation, hepatic synthetic function, and the coagulation system.

In addition, baseline serum albumin level on ICU day 1 showed a significant inverse association with subsequent ΔALB. In other words, patients with lower baseline albumin were more likely to experience an increase in albumin over the next 3 days, whereas those with higher baseline albumin were more likely to show a decline. In patients who did not receive exogenous HAS, this pattern may reflect a degree of endogenous compensatory regulation. One possible explanation is that lower albumin levels trigger greater hepatic albumin synthesis in the absence of exogenous supplementation.

Regarding nutritional support, we used enteral nutrition volume as a standardized and quantifiable indicator of nutritional input. We found no significant association between enteral nutrition volume during the first 3 ICU days and ΔALB over the same interval. This observation is consistent with earlier reports.[6-8] Hypoalbuminemia has historically been regarded as a marker of malnutrition that can be corrected by nutritional supplementation alone. However, contemporary evidence suggests that serum albumin is more closely linked to the physiological response to illness and inflammation than to nutritional intake per se.[2] Declines in albumin are commonly observed in the acute phase of illness, including after surgery and trauma.[2,9,10] The lack of association between nutritional support and albumin change in our study may also be consistent with the view that aggressive early nutrition does not necessarily translate into immediate biochemical recovery during acute critical illness.[6]

In the multivariable linear regression analysis, standardized coefficients (β) were used to compare the relative strength of association between each independent variable and ΔALB. Baseline serum albumin on day 1 showed the largest standardized coefficient (β=-0.359), supporting the possibility that endogenous compensatory mechanisms contribute substantially to early albumin dynamics. The associations of ΔHCT (β=0.254) and intravenous crystalloid volume (β=-0.176) with ΔALB suggest that hemodilution and hemoconcentration are major contributors to short-term albumin fluctuation. The association between ΔPLT and ΔALB (β=0.122) further suggests that platelet dynamics and albumin dynamics may track similar inflammatory processes early in sepsis. Finally, the inverse association between baseline ALT and ΔALB (β=-0.103) highlights the potential importance of early liver dysfunction as a determinant of impaired albumin recovery.

**References**

1. Wiedermann CJ. Hypoalbuminemia as Surrogate and Culprit of Infections. INT J MOL SCI 2021; 22(9).

2. Soeters PB, Wolfe RR, Shenkin A. Hypoalbuminemia: Pathogenesis and Clinical Significance. JPEN-PARENTER ENTER 2019; 43(2): 181-93.

3. Omiya K, Sato H, Sato T, et al. Albumin and fibrinogen kinetics in sepsis: a prospective observational study. CRIT CARE 2021; 25(1): 436.

4. Barle H, Hammarqvist F, Westman B, et al. Synthesis rates of total liver protein and albumin are both increased in patients with an acute inflammatory response. CLIN SCI 2006; 110(1): 93-9.

5. Verbruggen SC, Schierbeek H, Coss-Bu J, Joosten KF, Castillo L, van Goudoever JB. Albumin synthesis rates in post-surgical infants and septic adolescents; influence of amino acids, energy, and insulin. CLIN NUTR 2011; 30(4): 469-77.

6. Casaer MP, Mesotten D, Hermans G, et al. Early versus late parenteral nutrition in critically ill adults. NEW ENGL J MED 2011; 365(6): 506-17.

7. Van Dyck L, Casaer MP, Gunst J. Autophagy and Its Implications Against Early Full Nutrition Support in Critical Illness. NUTR CLIN PRACT 2018; 33(3): 339-47.

8. Pardo E, Lescot T, Preiser JC, et al. Association between early nutrition support and 28-day mortality in critically ill patients: the FRANS prospective nutrition cohort study. CRIT CARE 2023; 27(1): 7.

9. Mansoor O, Cayol M, Gachon P, et al. Albumin and fibrinogen syntheses increase while muscle protein synthesis decreases in head-injured patients. Am J Physiol 1997; 273(5): E898-902.

10. Kudsk KA, Tolley EA, DeWitt RC, et al. Preoperative albumin and surgical site identify surgical risk for major postoperative complications. JPEN-PARENTER ENTER 2003; 27(1): 1-9.

| **Table S1: Baseline characteristics and missing data in 12,128 patients with sepsis and available baseline serum albumin levels on ICU day 1 in the MIMIC-IV database.** | | |
| --- | --- | --- |
| Variables | N=12,128 | Missing, n (%) |
| Age | 66.4 (54.8, 78.5) | 0 (0) |
| Male (%) | 6876 (56.7) | 0 (0) |
| Weight (kg) | 78.0 (66.0, 93.6) | 447 (3.6) |
| Interventions, n (%) |  |  |
| RRT use (1^st^ 24 h) | 843 (7.0) | 0 (0) |
| MV use (1^st^ 24 h) | 5565 (45.9) | 0 (0) |
| Comorbidities, n (%) |  |  |
| CHF | 3599 (29.7) | 0 (0) |
| COPD | 3081 (25.4) | 0 (0) |
| Diabetes | 3710 (30.6) | 0 (0) |
| Malignancy | 1921 (15.8) | 0 (0) |
| AKI | 8297 (68.4) | 0 (0) |
| Vital signs |  |  |
| Heart rate (bpm) | 87.7 (76.3, 100.0) | 13 (< 0.1) |
| MAP (mmHg) | 75.5 (69.6, 83.0) | 13 (< 0.1) |
| Respiratory rate (bpm) | 19.6 (17.1, 22.7) | 18 (0.1) |
| Temperature (°C) | 36.9 (36.6, 37.2) | 301 (2.4) |
| Laboratory tests |  |  |
| Platelet (×10^9^/L) | 157.0 (101.0, 227.0) | 9 (< 0.1) |
| WBC (×10^9^/L) | 13.7 (9.4, 19.3) | 7 (< 0.1) |
| HCT (%) | 31.8 (27.3, 36.5) | 12 (< 0.1) |
| ALT (IU/L) | 34.0 (18.0, 84.0) | 612 (5.0) |
| Creatinine (mg/dL) | 1.3 (0.9, 2.1) | 5 (< 0.1) |
| Albumin (g/dL) | 3.1 (2.6, 3.6) | 0 (0) |
| Lactate level (mmol/L) | 2.4 (1.8, 3.6) | 3,531 (29.1) |
| Data are presented as median (interquartile range) for continuous variables and n (%) for categorical variables. “Missing” indicates the number and proportion of missing observations for each variable. Baseline serum albumin was defined as the lowest serum albumin concentration measured on ICU day 1.  Abbreviations: ICU, intensive care unit; MIMIC-IV, Medical Information Mart for Intensive Care, version IV; RRT, renal replacement therapy; MV, mechanical ventilation; CHF, congestive heart failure; COPD, chronic obstructive pulmonary disease; AKI, acute kidney injury; MAP, mean arterial pressure; WBC, white blood cell; HCT, hematocrit; ALT, alanine aminotransferase. | | |

| **Table S2: Complete multivariable Cox model outputs for baseline serum albumin and prognosis in patients with sepsis in the MIMIC-IV cohort.** | | | |
| --- | --- | --- | --- |
| **Variables** | **In-hospital outcome HR (95% CI, P value)** | **90-day outcome HR (95% CI, P value)** | **Overall outcome HR (95% CI, P value)** |
| Baseline serum albumin, RCS global test | P<0.001 | P<0.001 | P<0.001 |
| Baseline serum albumin, RCS nonlinearity test | P=0.004 | P<0.001 | P=0.002 |
| Age | 1.02 (1.02–1.03, P<0.001) | 1.02 (1.02–1.03, P<0.001) | 1.02 (1.02–1.02, P<0.001) |
| Male (%) | 1.00 (0.92–1.09, P=0.972) | 1.10 (1.03–1.18, P=0.005) | 1.13 (1.07–1.20, P<0.001) |
| Weight (kg) | 1.00 (1.00–1.00, P=0.015) | 0.99 (0.99–1.00, P<0.001) | 0.99 (0.99–1.00, P<0.001) |
| Interventions, n (%) |  |  |  |
| RRT use (1st 24 h) | 0.91 (0.78–1.05, P=0.207) | 1.10 (0.97–1.25, P=0.140) | 1.14 (1.02–1.26, P=0.020) |
| MV use (1st 24 h) | 1.37 (1.25–1.51, P<0.001) | 1.26 (1.18–1.35, P<0.001) | 1.07 (1.01–1.13, P=0.024) |
| Comorbidities, n (%) |  |  |  |
| CHF | 0.93 (0.85–1.02, P=0.116) | 1.08 (1.01–1.16, P=0.034) | 1.14 (1.08–1.21, P<0.001) |
| COPD | 1.03 (0.94–1.13, P=0.552) | 1.00 (0.93–1.07, P=0.922) | 1.11 (1.05–1.17, P=0.001) |
| Diabetes | 0.88 (0.80–0.96, P=0.007) | 0.93 (0.86–1.00, P=0.040) | 1.05 (1.00–1.11, P=0.071) |
| Malignancy | 1.24 (1.12–1.37, P<0.001) | 1.58 (1.46–1.71, P<0.001) | 1.75 (1.65–1.87, P<0.001) |
| AKI | 1.79 (1.57–2.04, P<0.001) | 1.74 (1.60–1.90, P<0.001) | 1.39 (1.31–1.48, P<0.001) |
| Vital signs |  |  |  |
| Heart rate (bpm) | 1.01 (1.01–1.01, P<0.001) | 1.01 (1.01–1.01, P<0.001) | 1.01 (1.00–1.01, P<0.001) |
| MAP (mmHg) | 0.98 (0.98–0.99, P<0.001) | 0.99 (0.98–0.99, P<0.001) | 0.99 (0.99–0.99, P<0.001) |
| Respiratory rate (bpm) | 1.06 (1.05–1.07, P<0.001) | 1.05 (1.04–1.05, P<0.001) | 1.03 (1.03–1.04, P<0.001) |
| Temperature (°C) | 0.67 (0.63–0.70, P<0.001) | 0.66 (0.63–0.69, P<0.001) | 0.68 (0.65–0.71, P<0.001) |
| Laboratory tests |  |  |  |
| Platelet (×10⁹/L) | 1.00 (1.00–1.00, P=0.031) | 1.00 (1.00–1.00, P=0.094) | 1.00 (1.00–1.00, P=0.035) |
| WBC (×10⁹/L) | 1.00 (1.00–1.00, P=0.122) | 1.00 (1.00–1.00, P=0.011) | 1.00 (1.00–1.00, P=0.385) |
| HCT (%) | 1.01 (1.00–1.02, P=0.005) | 1.00 (0.99–1.01, P=0.987) | 0.99 (0.99–1.00, P<0.001) |
| ALT (IU/L) | 1.00 (1.00–1.00, P=0.254) | 1.00 (1.00–1.00, P=0.933) | 1.00 (1.00–1.00, P=0.006) |
| Creatinine (mg/dL) | 1.05 (1.03–1.08, P<0.001) | 1.04 (1.02–1.06, P<0.001) | 1.04 (1.02–1.06, P<0.001) |
| Lactate level (mmol/L) | 1.05 (1.04–1.06, P<0.001) | 1.04 (1.04–1.05, P<0.001) | 1.04 (1.03–1.04, P<0.001) |
| Hazard ratios for continuous variables are expressed per one-unit increase. For binary variables, the reference category is absence of the corresponding condition or intervention. Baseline serum albumin was defined as the lowest serum albumin concentration measured on ICU day 1. Serum albumin was modeled using restricted cubic splines in the primary analysis shown in Figure 2; the RCS global and nonlinearity tests are shown at the top of this table.  Abbreviations: HR, hazard ratio; CI, confidence interval; ICU, intensive care unit; RRT, renal replacement therapy; MV, mechanical ventilation; CHF, congestive heart failure; COPD, chronic obstructive pulmonary disease; AKI, acute kidney injury; MAP, mean arterial pressure; WBC, white blood cell; HCT, hematocrit; ALT, alanine aminotransferase. | | | |

| **Table S3: Baseline characteristics and missing data in 3,084 patients with sepsis and available early serum albumin change (ΔALB) during the first 3 ICU days in the MIMIC-IV database.** | | |
| --- | --- | --- |
| Variables | N=3,084 | Missing, n (%) |
| Age | 62.9 (52.2, 74.0) | 0 (0) |
| Male, n (%) | 1831 (59.4) | 0 (0) |
| Weight (kg) | 80.0 (68.0, 95.0) | 133 (4.3) |
| Interventions |  |  |
| RRT use (1^st^ 24h) | 297 (9.6) | 0 (0) |
| MV use (1^st^ 24h) | 1621 (52.6) | 0 (0) |
| Comorbidities |  |  |
| CHF | 782 (25.4) | 0 (0) |
| COPD | 693 (22.5) | 0 (0) |
| Diabetes | 894 (29.0) | 0 (0) |
| Malignancy | 540 (17.5) | 0 (0) |
| AKI | 2307 (74.8) | 0 (0) |
| Vital signs |  |  |
| Heart rate (bpm) | 89.0 (77.5, 101.9) | 6 (0.1) |
| MAP (mmHg) | 75.8 (69.8, 83.1) | 6 (0.1) |
| Respiratory rate (bpm) | 19.6 (17.0, 22.7) | 7 (0.2) |
| Temperature (°C) | 36.9 (36.6, 37.2) | 99 (3.2) |
| Laboratory tests |  |  |
| Platelet (×10^9^/L) | 128.0 (72.0, 202.0) | 3 (< 0.1) |
| WBC (×10^9^/L) | 13.9 (9.4, 19.8) | 3 (< 0.1) |
| HCT (%) | 30.9 (26.7, 35.9) | 0 (0) |
| ALT (IU/L) | 52.0 (24.0, 162.0) | 196 (6.3) |
| Creatinine (mg/dL) | 1.4 (0.9, 2.4) | 2 (< 0.1) |
| Albumin (g/dL) | 2.8 (2.4, 3.2) | 180 (5.8) |
| Lactate level (mmol/L) | 2.7 (1.8, 4.5) | 630 (20.4) |
| ΔHCT | -1.7 (-5.2, 1.2) | 3 (< 0.1) |
| ΔALB | -0.1(-0.4, 0.1) | 0 (0) |
| Data are presented as median (interquartile range) for continuous variables and n (%) for categorical variables. “Missing” indicates the number and proportion of missing observations for each variable. ΔALB was defined as the change in serum albumin concentration during the first 3 days after ICU admission, calculated as the latest value minus the earliest value.  Abbreviations: ICU, intensive care unit; MIMIC-IV, Medical Information Mart for Intensive Care, version IV; ΔALB, change in serum albumin during the first 3 ICU days; RRT, renal replacement therapy; MV, mechanical ventilation; CHF, congestive heart failure; COPD, chronic obstructive pulmonary disease; AKI, acute kidney injury; MAP, mean arterial pressure; WBC, white blood cell; HCT, hematocrit; ALT, alanine aminotransferase; INR, international normalized ratio. | | |

| **Table S4: Baseline characteristics and missing data in 689 patients with sepsis and available early serum albumin change (ΔALB) during the first 3 ICU days in the First Affiliated Hospital of Xi'an Jiaotong University cohort.** | | |
| --- | --- | --- |
| Variables | N=689 | Missing, n (%) |
| Age | 62.0 (50.0,74.0) | 0 (0) |
| Male (%) | 441 (64.0) | 0 (0) |
| Interventions, n (%) |  |  |
| RRT use (1^st^ 24 h) | 4 (0.6) | 0 (0) |
| MV use (1^st^ 24 h) | 101 (14.7) | 0 (0) |
| Comorbidities, n (%) |  |  |
| CHF | 19 (2.8) | 0 (0) |
| COPD | 41 (6.0) | 0 (0) |
| Diabetes | 186 (27.0) | 0 (0) |
| Malignancy | 58 (8.4) | 0 (0) |
| AKI | 124 (18.0) | 0 (0) |
| Vital signs |  |  |
| Heart rate (bpm) | 98.2 (89.5, 108.2) | 110 (15.9) |
| MAP (mmHg) | 92.6 (85.5, 100.2) | 146 (21.1) |
| Respiratory rate (bpm) | 21.3 (18.5, 24.0) | 90 (13.0) |
| Temperature (°C) | 36.8 (36.5, 37.2) | 90 (13.0) |
| Laboratory tests |  |  |
| Platelet (×10^9^/L) | 82.0 (40.0, 132.0) | 26 (3.7) |
| WBC (×10^9^/L) | 12.7 (8.2, 19.9) | 26 (3.7) |
| HCT (%) | 29.9 (24.8, 35.0) | 25 (3.6) |
| AST (IU/L) | 57.0 (28.0, 146.0) | 27 (3.9) |
| BUN (mg/dL) | 14.8 (9.1, 21.8) | 27 (3.9) |
| Albumin (g/dL) | 2.7 (2.4, 3.0) | 28 (4.0) |
| ΔHCT | -1.8 (-5.5, 2.1) | 4 (0.5) |
| ΔALB | 0.2 (-0.1, 0.5) | 0 (0) |
| Data are presented as median (interquartile range) for continuous variables and n (%) for categorical variables. “Missing” indicates the number and proportion of missing observations for each variable. ΔALB was defined as the change in serum albumin concentration during the first 3 days after ICU admission, calculated as the latest value minus the earliest value.  Abbreviations: ICU, intensive care unit; ΔALB, change in serum albumin during the first 3 ICU days; HAS, human albumin solution; RRT, renal replacement therapy; MV, mechanical ventilation; CHF, congestive heart failure; COPD, chronic obstructive pulmonary disease; AKI, acute kidney injury; MAP, mean arterial pressure; WBC, white blood cell; HCT, hematocrit; AST, aspartate aminotransferase; BUN, blood urea nitrogen. | | |

| **Table S5: Baseline characteristics and missing data in 2,173 patients with sepsis, available ΔALB, and no human albumin solution exposure in the MIMIC-IV database.** | | | |
| --- | --- | --- | --- |
| Variables | N=2,173 | Missing, n (%) | |
| ΔALB | -0.2 (-0.4, 0.0) | 0 (0) | |
| Age | 63.7 (52.1, 75.6) | 0 (0) | |
| Male, n (%) | 1253 (57.7) | 0 (0) | |
| Weight (kg) | 78.9 (66.6, 93.8) | 77 (3.5) | |
| Interventions |  |  | |
| RRT use (1^st^ 24h) | 180 (8.3) | 0 (0) | |
| MV use (1^st^ 24h) | 1038 (47.8) | 0 (0) | |
| Comorbidities |  |  | |
| CHF | 597 (27.5) | 0 (0) | |
| COPD | 502 (23.1) | 0 (0) | |
| Diabetes | 647 (29.8) | 0 (0) | |
| Malignancy | 378 (17.4) | 0 (0) | |
| AKI | 1494 (68.8) | 0 (0) | |
| Vital signs |  |  | |
| Heart rate (bpm) | 88.2 (76.4, 101.1) | 5 (0.2) | |
| MAP (mmHg) | 77.0 (70.7, 84.7) | 5 (0.2) | |
| Respiratory rate (bpm) | 19.8 (17.2, 22.9) | 6 (0.2) | |
| Temperature (°C) | 36.9 (36.6, 37.3) | 40 (1.8) | |
| ΔHeart rate | -6.2 (-20.0, 5.0) | 197 (9.0) | |
| ΔMAP | -2.0 (-14.0, 10.0) | 201 (9.2) | |
| ΔRespiratory rate | 0.0 (-5.0, 4.0) | 196 (9.0) | |
| ΔTemperature | 0.0 (-0.4, 0.5) | 274 (12.6) | |
| Laboratory tests |  |  | |
| Platelet (×10^9^/L) | 148.0 (89.0, 218.0) | 2 (< 0.1) | |
| WBC (×10^9^/L) | 13.5 (9.1, 19.6) | 2 (< 0.1) | |
| HCT (%) | 31.5 (27.4, 36.3) | 0 (0) | |
| ALT (IU/L) | 52.0 (24.0, 148.0) | 167 (7.6) | |
| Creatinine (mg/dL) | 1.3 (0.9, 2.2) | 0 (0) | |
| Albumin (g/dL) | 2.9 (2.5, 3.3) | 125 (5.7) | |
| Lactate level (mmol/L) | 2.4 (1.7, 3.7) | 521 (23.9) | |
| Blood glucose (mmol/L) | 7.3 (5.9, 9.9) | 0 (0) | |
| INR | 1.4 (1.2, 1.8) | 75 (3.4) | |
| ΔPlatelet | -14.0 (-50.0, 14.0) | 10 (0.4) | |
| ΔWBC | -1.4 (-4.8, 1.4) | 9 (0.4) | |
| ΔHCT | -1.7 (-4.9, 0.9) | 9 (0.4) | |
| ΔALT | -4.8 (-28.9, 3.0) | 313 (14.4) | |
| ΔCreatinine | -0.1 (-0.3, 0.1) | 5 (0.2) | |
| ΔBlood glucose | -0.7 (-2.7, 0.8) | 3 (0.1) | |
| ΔINR | -0.1 (-0.2, 0.0) | 306 (14.0) | |
| Input and output events |  |  | |
| Urine output (ml) (1^st^ 24h) | 1495.0 (873.0, 2276.0) | 65 (2.9) | |
| Intravenous crystalloid input (ml) (1^st^ 72 hours) | 4425.9 (2184.3, 7557.7) | 0 (0) | |
| Enteral nutrition (ml) (1^st^ 72 hours) | 509.7 (208.8, 1150.0)* | 0 (0) | |
| ΔALB serum albumin change in the first 3 days of ICU, HAS human albumin solution, RRT renal replacement therapy, MV mechanical ventilation, CHF congestive heart failure, COPD chronic obstructive pulmonary disease, AKI acute kidney injury, MAP mean arterial pressure, WBC white blood cell, HCT hematocrit, ALT alanine aminotransferase, "Δxx" Changes of this laboratory parameter in the first 3 days after admission to ICU  * Median and quartiles of enteral nutrition are presented for 492 of the 2173 patients who received enteral nutrition within 3 days of ICU admission. | | |  |

| **Table S6: Multivariable linear regression analysis of factors associated with endogenous ΔALB in patients with sepsis, ordered by standardized coefficient (β).** | | | | | | | |
| --- | --- | --- | --- | --- | --- | --- | --- |
| Dependent variable：  ΔALB | Unstandardized Coefficients  (B) | Standardized Coefficients  (β) | P | B: 95.0% CI | | Collinearity statistics Tolerance | VIF |
|  |  |  |  | Lower | Upper |  |  |
| Constant： | 1.657 |  | <0.001 | 0.802 | 2.512 |  |  |
| Albumin (g/dL) | -0.230 | -0.359 | <0.001 | -0.255 | -0.205 | 0.814 | 1.228 |
| ΔHCT | 0.019 | 0.254 | <0.001 | 0.015 | 0.023 | 0.484 | 2.066 |
| Intravenous crystalloid input  (ml) (1^st^ 72 hours) | 0.000 | -0.176 | <0.001 | 0.000 | 0.000 | 0.749 | 1.336 |
| ΔPlatelet | 0.001 | 0.122 | <0.001 | 0.000 | 0.001 | 0.850 | 1.177 |
| ALT (IU/L) | 0.000 | -0.103 | <0.001 | 0.000 | 0.000 | 0.709 | 1.409 |
| HCT (%) | 0.005 | 0.088 | 0.001 | 0.002 | 0.008 | 0.493 | 2.027 |
| Age | -0.002 | -0.080 | <0.001 | -0.003 | -0.001 | 0.766 | 1.305 |
| Urine output (ml) (1^st^ 24h) | 0.000 | 0.073 | <0.001 | 0.000 | 0.000 | 0.840 | 1.190 |
| ΔINR | -0.036 | -0.073 | <0.001 | -0.055 | -0.017 | 0.849 | 1.177 |
| Heart rate (bpm) | -0.002 | -0.069 | 0.001 | -0.003 | -0.001 | 0.752 | 1.330 |
| ΔMAP | 0.001 | 0.069 | <0.001 | 0.001 | 0.002 | 0.943 | 1.060 |
| ΔALT | 0.000 | -0.066 | 0.002 | 0.000 | 0.000 | 0.724 | 1.381 |
| MAP (mmHg) | 0.002 | 0.059 | 0.002 | 0.001 | 0.003 | 0.859 | 1.164 |
| RRT use (1^st^ 24h) | 0.078 | 0.054 | 0.005 | 0.023 | 0.133 | 0.846 | 1.183 |
| Temperature (°C) | -0.029 | -0.048 | 0.013 | -0.052 | -0.006 | 0.857 | 1.166 |
| Respiratory rate (bpm) | -0.004 | -0.043 | 0.028 | -0.007 | 0.000 | 0.828 | 1.207 |
| *R^2^=0.308, adjusted R^2^=0.303  ΔALB serum albumin change in the first 3 days of ICU, "Δxx" Changes of this laboratory parameter in the first 3 days after admission to ICU, HAS human albumin solution, RRT renal replacement therapy, MV mechanical ventilation, CHF congestive heart failure, COPD chronic obstructive pulmonary disease, AKI acute kidney injury, MAP mean arterial pressure, WBC white blood cell, HCT hematocrit, ALT alanine aminotransferase, VIF Variance Inflation Factor | | | | | | | |

| **Table S7: Changes in serum albumin before and after each HAS administration in the MIMIC-IV database.** | | | | | | | | | | | |
| --- | --- | --- | --- | --- | --- | --- | --- | --- | --- | --- | --- |
|  | Within 24 hours after infusion (n=1,576) | | | | 72 hours after infusion (n=968) | | | | 7 days after infusion (n=578) | | |
|  | Original | | HCT_Corrected | | Original | | HCT_Corrected | | Original | | HCT_Corrected |
| Albumin (g/dL) | 3.003 | | 3.217 | | 2.778 | | 2.890 | | 2.652 | | 2.792 |
| Δalb (g/dL) | 0.159 | | 0.373 | | -0.044 | | 0.069 | | -0.110 | | 0.030 |
| 95% CI | 0.135 — 0.183 | | 0.335 — 0.411 | | -0.076 — -0.012 | | 0.023 — 0.115 | | -0.158 — -0.063 | | -0.036 — 0.097 |
| P-value | <0.001 | | <0.001 | | 0.007 | | 0.003 | | <0.001 | | 0.375 |
| Δalb Changes of serum albumin levels before and after human albumin infusion | | | | | | | | | | | |
|  |  |  | |  | |  | |  | |  | |
| **Table S8: Changes in serum albumin before and after each HAS administration in the same MIMIC-IV cohort.** | | | | | | | | | | | |
|  | Within 24 hours after infusion | | | | 72 hours after infusion | | | | 7 days after infusion | | |
| n=218 | Original | | HCT_Corrected | | Original | | HCT_Corrected | | Original | | HCT_Corrected |
| Albumin(g/dL) | 3.017 | | 3.255 | | 2.825 | | 2.938 | | 2.724 | | 2.832 |
| Δalb (g/dL) | 0.203 | | 0.441 | | 0.011 | | 0.124 | | -0.089 | | 0.018 |
| 95% CI | 0.141 — 0.266 | | 0.337 — 0.546 | | -0.057 — 0.080 | | 0.030— 0.218 | | -0.164 — -0.014 | | -0.094 — 0.131 |
| P-value | <0.001 | | <0.001 | | 0.741 | | 0.010 | | 0.020 | | 0.749 |
| Δalb Changes of serum albumin levels before and after human albumin infusion | | | | | | | | | | | |

| **Table S9: Changes in serum albumin before and after all HAS administrations per patient in the MIMIC-IV database.** | | | | | | | | | | | |
| --- | --- | --- | --- | --- | --- | --- | --- | --- | --- | --- | --- |
|  | Within 24 hours after infusion (n=652) | | | | 72 hours after infusion (n=655) | | | | 7 days after infusion (n=568) | | |
|  | Original | | HCT_Corrected | | Original | | HCT_Corrected | | Original | | HCT_Corrected |
| Albumin (g/dL) | 3.010 | | 3.489 | | 2.820 | | 3.217 | | 2.707 | | 3.201 |
| Δalb (g/dL) | 0.379 | | 0.858 | | 0.149 | | 0.546 | | 0.091 | | 0.585 |
| 95% CI | 0.325 — 0.433 | | 0.769 — 0.948 | | 0.099 — 0.199 | | 0.463 — 0.628 | | 0.034 — 0.148 | | 0.495 — 0.675 |
| P-value | <0.001 | | <0.001 | | <0.001 | | <0.001 | | 0.002 | | <0.001 |
| Δalb Changes of serum albumin levels before and after human albumin infusion | | | | | | | | | | | |
|  |  |  | |  | |  | |  | |  | |
| **Table S10: Changes in serum albumin before and after all HAS administrations per patient in the same MIMIC-IV cohort.** | | | | | | | | | | | |
|  | Within 24 hours after infusion | | | | 72 hours after infusion | | | | 7 days after infusion | | |
| n=137 | Original | | HCT_Corrected | | Original | | HCT_Corrected | | Original | | HCT_Corrected |
| Albumin(g/dL) | 3.061 | | 3.645 | | 2.875 | | 3.289 | | 2.796 | | 3.187 |
| Δalb (g/dL) | 0.425 | | 1.009 | | 0.239 | | 0.652 | | 0.160 | | 0.551 |
| 95% CI | 0.299 — 0.551 | | 0.793 — 1.224 | | 0.117 — 0.361 | | 0.449 — 0.856 | | 0.037 — 0.283 | | 0.349 — 0.753 |
| P-value | <0.001 | | <0.001 | | <0.001 | | <0.001 | | 0.011 | | <0.001 |
| Δalb Changes of serum albumin levels before and after human albumin infusion | | | | | | | | | | | |

| **Table S11: Changes in serum albumin before and after each HAS administration in the First Affiliated Hospital of Xi'an Jiaotong University cohort.** | | | | | | | | | | | |
| --- | --- | --- | --- | --- | --- | --- | --- | --- | --- | --- | --- |
|  | Within 24 hours after infusion (n=3,565) | | | 72 hours after infusion (n=381) | | | | | 7 days after infusion (n=101) | | |
|  | Original | HCT_Corrected | | Original | | HCT_Corrected | | | Original | | HCT_Corrected |
| Albumin (g/dL) | 2.979 | 3.011 | | 3.064 | | 3.105 | | | 3.250 | | 3.293 |
| Δalb (g/dL) | 0.036 | 0.068 | | 0.030 | | 0.071 | | | 0.181 | | 0.225 |
| 95% CI | 0.023 — 0.049 | 0.048 — 0.088 | | -0.017 — 0.077 | | 0.005 — 0.138 | | | 0.083 — 0.280 | | 0.047 — 0.402 |
| P-value | <0.001 | <0.001 | | 0.208 | | 0.035 | | | <0.001 | | 0.014 |
| Δalb Changes of serum albumin levels before and after human albumin infusion | | | | | | | | | | | |
|  |  | |  | |  | |  |  | |  | |
| **Table S12: Changes in serum albumin before and after each HAS administration in the same external validation cohort.** | | | | | | | | | | | |
|  | Within 24 hours after infusion | | | 72 hours after infusion | | | | | 7 days after infusion | | |
| n=79 | Original | HCT_Corrected | | Original | | HCT_Corrected | | | Original | | HCT_Corrected |
| Albumin(g/dL) | 3.209 | 3.239 | | 3.204 | | 3.156 | | | 3.237 | | 3.244 |
| Δalb (g/dL) | 0.124 | 0.154 | | 0.119 | | 0.071 | | | 0.152 | | 0.159 |
| 95% CI | 0.057 — 0.191 | 0.030 — 0.279 | | 0.043 — 0.195 | | -0.047— 0.189 | | | 0.039 — 0.265 | | -0.028 — 0.346 |
| P-value | <0.001 | 0.016 | | 0.003 | | 0.234 | | | 0.009 | | 0.094 |
| Δalb Changes of serum albumin levels before and after human albumin infusion | | | | | | | | | | | |

| **Table S13: Changes in serum albumin before and after all HAS administrations per patient in the First Affiliated Hospital of Xi'an Jiaotong University cohort.** | | | | | | |
| --- | --- | --- | --- | --- | --- | --- |
|  | Within 24 hours after infusion (n=196) | | 72 hours after infusion (n=83) | | 7 days after infusion (n=41) | |
|  | Original | HCT_Corrected | Original | HCT_Corrected | Original | HCT_Corrected |
| Albumin (g/dL) | 3.046 | 3.450 | 3.075 | 3.247 | 3.171 | 3.401 |
| Δalb (g/dL) | 0.259 | 0.663 | 0.289 | 0.462 | 0.405 | 0.636 |
| 95% CI | 0.173 — 0.345 | 0.497 — 0.830 | 0.185 — 0.394 | 0.238 — 0.685 | 0.241 — 0.569 | 0.258 — 1.013 |
| P-value | <0.001 | <0.001 | <0.001 | <0.001 | <0.001 | 0.002 |
| Δalb Changes of serum albumin levels before and after human albumin infusion | | | | | | |
|  | | | | | | |
| **Table S14: Changes in serum albumin before and after all HAS administrations per patient in the same external validation cohort.** | | | | | | |
|  | Within 24 hours after infusion | | 72 hours after infusion | | 7 days after infusion | |
| n=28 | Original | HCT_Corrected | Original | HCT_Corrected | Original | HCT_Corrected |
| Albumin(g/dL) | 3.164 | 3.399 | 3.050 | 3.298 | 3.189 | 3.478 |
| Δalb (g/dL) | 0.371 | 0.606 | 0.257 | 0.505 | 0.396 | 0.685 |
| 95% CI | 0.174 — 0.569 | 0.190 — 1.022 | 0.075 — 0.439 | 0.129 — 0.882 | 0.189 — 0.603 | 0.297 — 1.072 |
| P-value | 0.001 | 0.006 | 0.007 | 0.010 | 0.001 | 0.001 |
| Δalb Changes of serum albumin levels before and after human albumin infusion | | | | | | |

| **Table S15. Timing of serum albumin measurements relative to HAS administration among MIMIC-IV HAS recipients with calculable ΔALB.** | | | |
| --- | --- | --- | --- |
| **Timing category** | **Definition** | **N** | **%** |
| All HAS-treated patients with calculable ΔALB | Original HAS-exposed ΔALB cohort | 911 | 100.0 |
| Pre-HAS ΔALB | latest albumin measurement occurred before or at first HAS start time | 37 | 4.1 |
| Excluding pre-HAS ΔALB | ΔALB not entirely before first HAS administration | 874 | 95.9 |
| Latest albumin after any HAS completion | latest albumin measurement occurred after completion of at least one HAS infusion | 752 | 82.5 |
| Strict pre-post HAS | first albumin before first HAS start and latest albumin after first HAS stop | 470 | 51.6 |
| HAS exposure was defined as at least one HAS infusion with stop time within 72 hours after ICU admission. ΔALB was calculated as the latest minus earliest serum albumin value during the first 72 hours after ICU admission, with the two measurements at least 24 hours apart. | | | |

| **Table S16. Sensitivity analysis for the association between baseline serum albumin and prognosis after excluding potentially post-baseline AKI from the multivariable Cox models** | | | | |
| --- | --- | --- | --- | --- |
| **Outcome** | **Original model P-overall** | **Original P-nonlinearity** | **Model excluding AKI P-overall** | **Model excluding AKI P-nonlinearity** |
| In-hospital outcome | <0.001 | 0.004 | <0.001 | 0.002 |
| 90-day outcome | <0.001 | <0.001 | <0.001 | <0.001 |
| Overall outcome | <0.001 | 0.002 | <0.001 | 0.001 |
| AKI, acute kidney injury; RCS, restricted cubic spline. Baseline serum albumin was modeled using RCS within multivariable Cox regression models. The primary model included AKI as an early organ dysfunction covariate. Because AKI within the first 3 ICU days may occur after baseline serum albumin measurement, sensitivity analyses were performed after excluding AKI from the adjustment set. P-overall represents the global association test, and P-nonlinearity represents the test for departure from linearity. | | | | |

| **Table S17. Interaction between baseline serum albumin level and ΔALB for 90-day prognosis in the MIMIC-IV cohort.** | | | | | | |
| --- | --- | --- | --- | --- | --- | --- |
| **Cohort** | **Baseline albumin stratum** | **n** | **Events** | **HR per 1-g/dL increase in ΔALB (95% CI)** | **P value** | **P-interaction** |
| Non-HAS | <2.77 g/dL | 862 | 323 | 0.51 (0.37–0.71) | <0.001 | 0.29 |
| Non-HAS | ≥2.77 g/dL | 1,311 | 328 | 0.59 (0.44–0.80) | <0.001 |  |
| HAS | <2.77 g/dL | 528 | 204 | 1.08 (0.85–1.37) | 0.545 | 0.67 |
| HAS | ≥2.77 g/dL | 383 | 150 | 1.00 (0.76–1.32) | 0.985 |  |
| Baseline serum albumin was defined as the lowest serum albumin concentration measured on ICU day 1 and was dichotomized using the 2.77 g/dL threshold identified in the baseline albumin restricted cubic spline analysis. Hazard ratios represent the association between ΔALB and 90-day mortality risk per 1-g/dL increase in serum albumin during the first 3 ICU days. Stratum-specific Cox models were adjusted for the same covariates as in the primary ΔALB analyses, including age, sex, body weight, congestive heart failure, chronic obstructive pulmonary disease, malignancy, diabetes, mean heart rate, mean arterial pressure, mean respiratory rate, mean body temperature, renal replacement therapy, mechanical ventilation, acute kidney injury, lowest platelet count, highest white blood cell count, baseline hematocrit, change in hematocrit, highest alanine aminotransferase, highest lactate, and highest creatinine. P-interaction values were obtained using likelihood ratio tests comparing Cox models with and without the ΔALB × baseline albumin category interaction term within each HAS-exposure group. Analyses were performed in the MIMIC-IV cohort; the external cohort was not used for this interaction analysis because only 19 patients were not exposed to HAS.  Abbreviations: ΔALB, change in serum albumin during the first 3 ICU days; HAS, human albumin solution; HR, hazard ratio; CI, confidence interval; ICU, intensive care unit. | | | | | | |


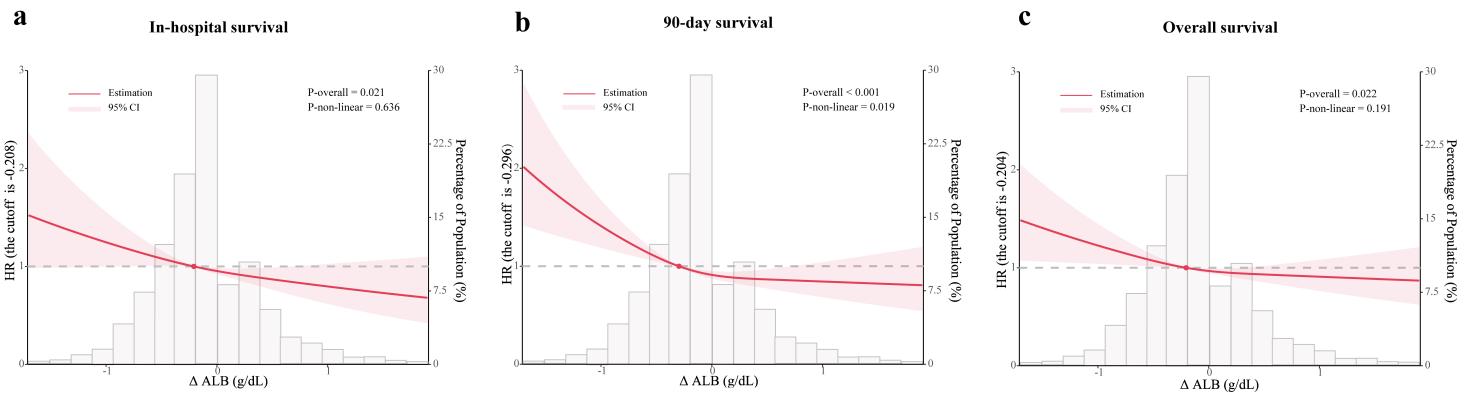


**Figure S1:** Association of early serum albumin change (ΔALB) with prognosis in patients with sepsis in the MIMIC-IV database (n=3,084).

(a) In-hospital survival.

(b) 90-day survival.

(c) Overall survival.

P-overall indicates the global test for association between ΔALB and the outcome, whereas P-nonlinearity indicates the test for departure from linearity. The spline curves were used to describe the adjusted exposure–response pattern and were not intended as formal segmented analyses above and below a specific cutoff.


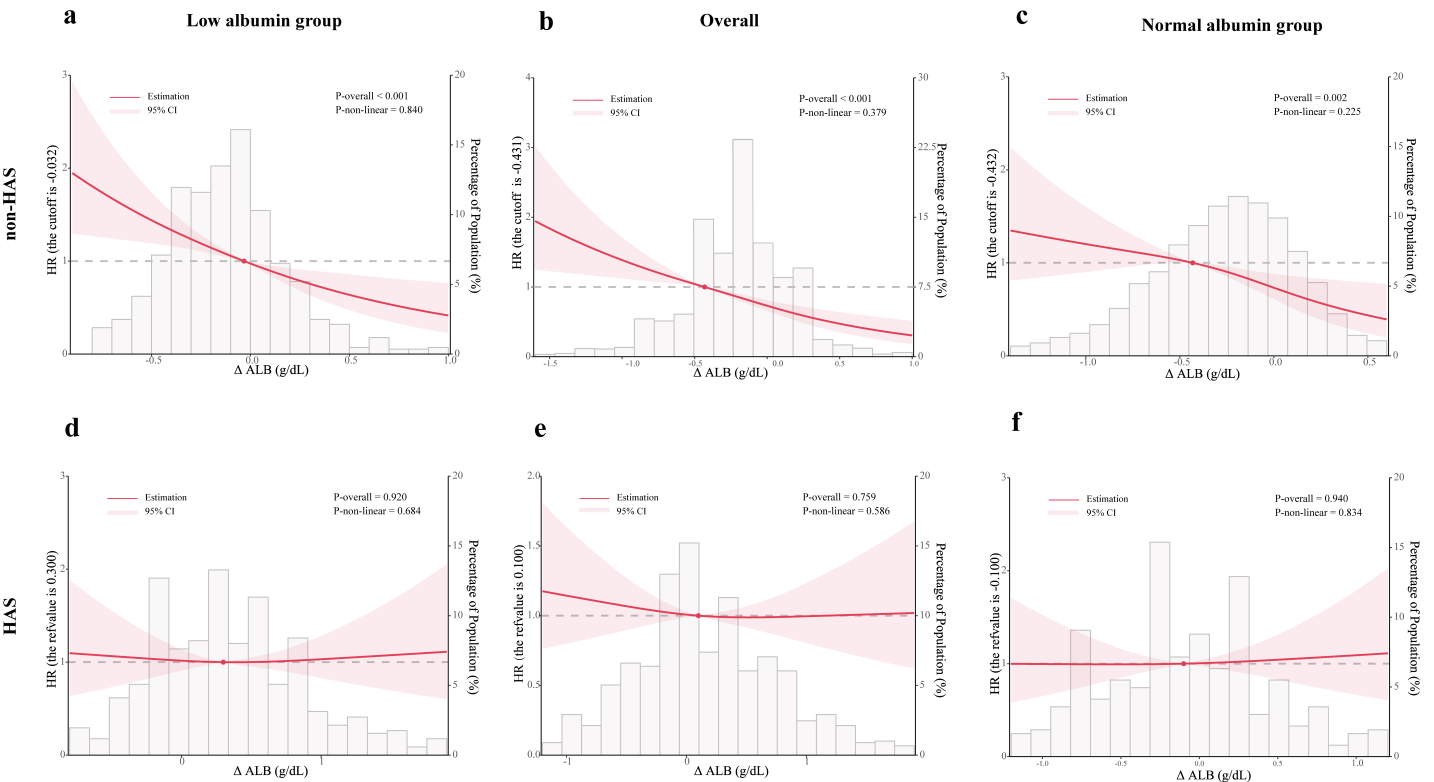


**Figure S2:** Association of early serum albumin change (ΔALB) with 90-day survival in sepsis, stratified by baseline serum albumin level and human albumin solution (HAS) use, in the MIMIC-IV database.

(a) Low baseline albumin group (<2.77 g/dL) without HAS use (n=862).

(b) Overall non-HAS cohort (n=2,173).

(c) Higher baseline albumin group (≥2.77 g/dL) without HAS use (n=1,311).

(d) Low baseline albumin group (<2.77 g/dL) with HAS use (n=528).

(e) Overall HAS cohort (n=911).

(f) Higher baseline albumin group (≥2.77 g/dL) with HAS use (n=383).


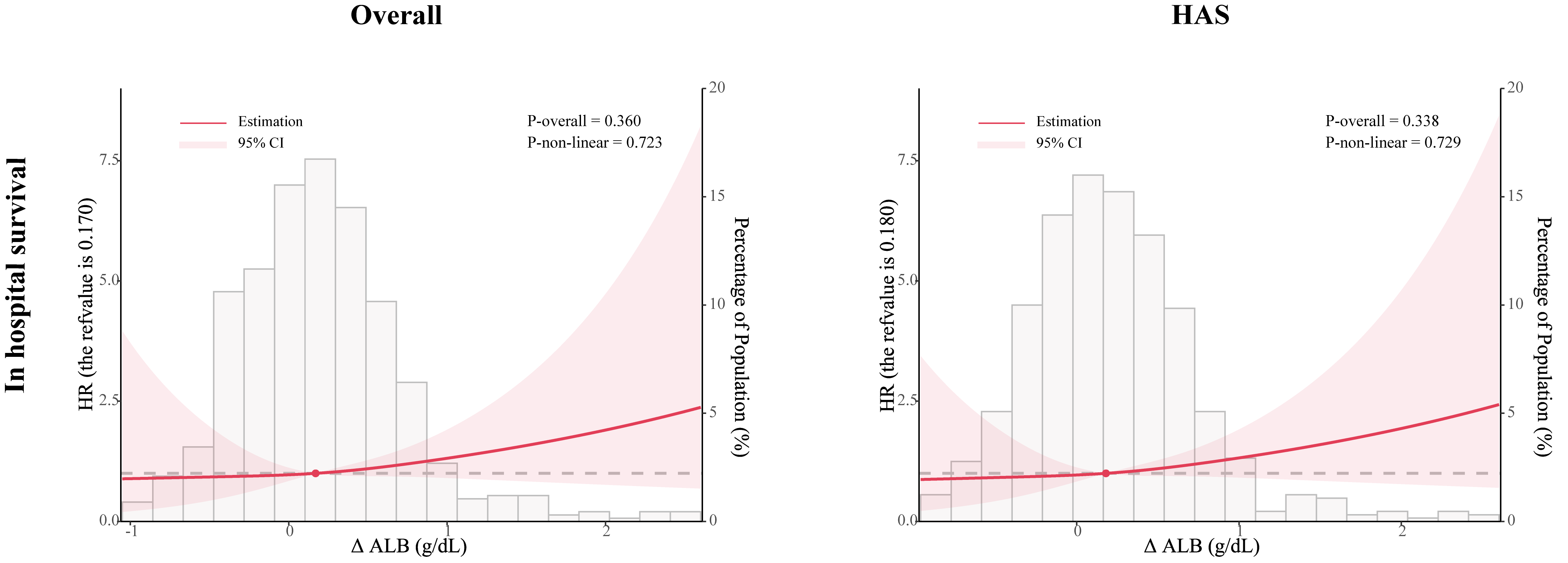


**Figure S3:** Association of early serum albumin change (ΔALB) with prognosis in patients with sepsis from the First Affiliated Hospital of Xi'an Jiaotong University, stratified by human albumin solution (HAS) use.

(a) Overall cohort (n=689).

(b) HAS cohort (n=670).


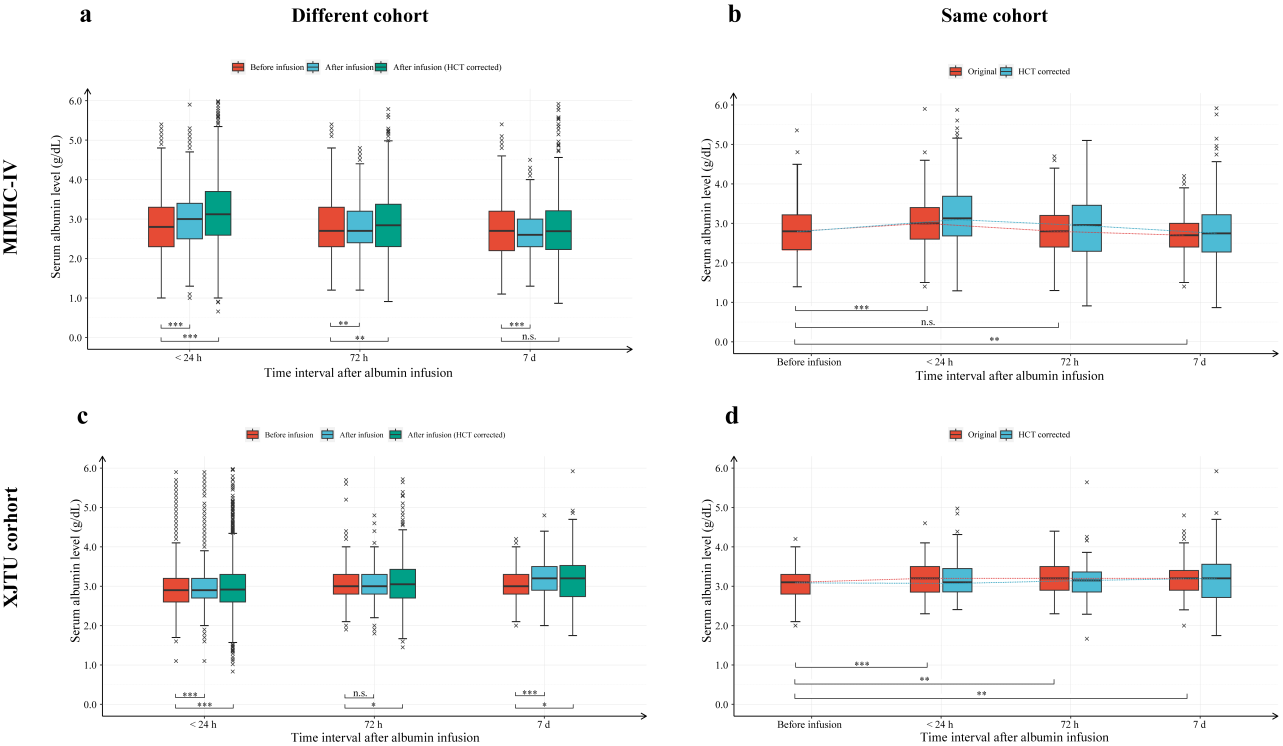


**Figure S4:** Effect of human albumin solution (HAS) administration on serum albumin levels in patients with sepsis, analyzed on a per-infusion basis.

(a) Serum albumin levels at different time points before and after HAS administration in the MIMIC-IV cohort with incomplete albumin measurements.

(b) Serum albumin levels at different time points before and after HAS administration in the MIMIC-IV cohort with complete albumin measurements (4 time points).

(c) Serum albumin levels at different time points before and after HAS administration in the First Affiliated Hospital of Xi'an Jiaotong University cohort with incomplete albumin measurements.

(d) Serum albumin levels at different time points before and after HAS administration in the First Affiliated Hospital of Xi'an Jiaotong University cohort with complete albumin measurements (4 time points).

Abbreviations: XJTU, the First Affiliated Hospital of Xi'an Jiaotong University.

Definitions: Before infusion, the most recent serum albumin measurement within 72 hours before the start of HAS administration; <24 h, the earliest serum albumin measurement within 24 hours after completion of HAS administration; 72 h, the earliest serum albumin measurement obtained 48-96 hours after completion of HAS administration; 7 days, the earliest serum albumin measurement obtained 6-14 days after completion of HAS administration; HCT-corrected, serum albumin corrected for hematocrit.


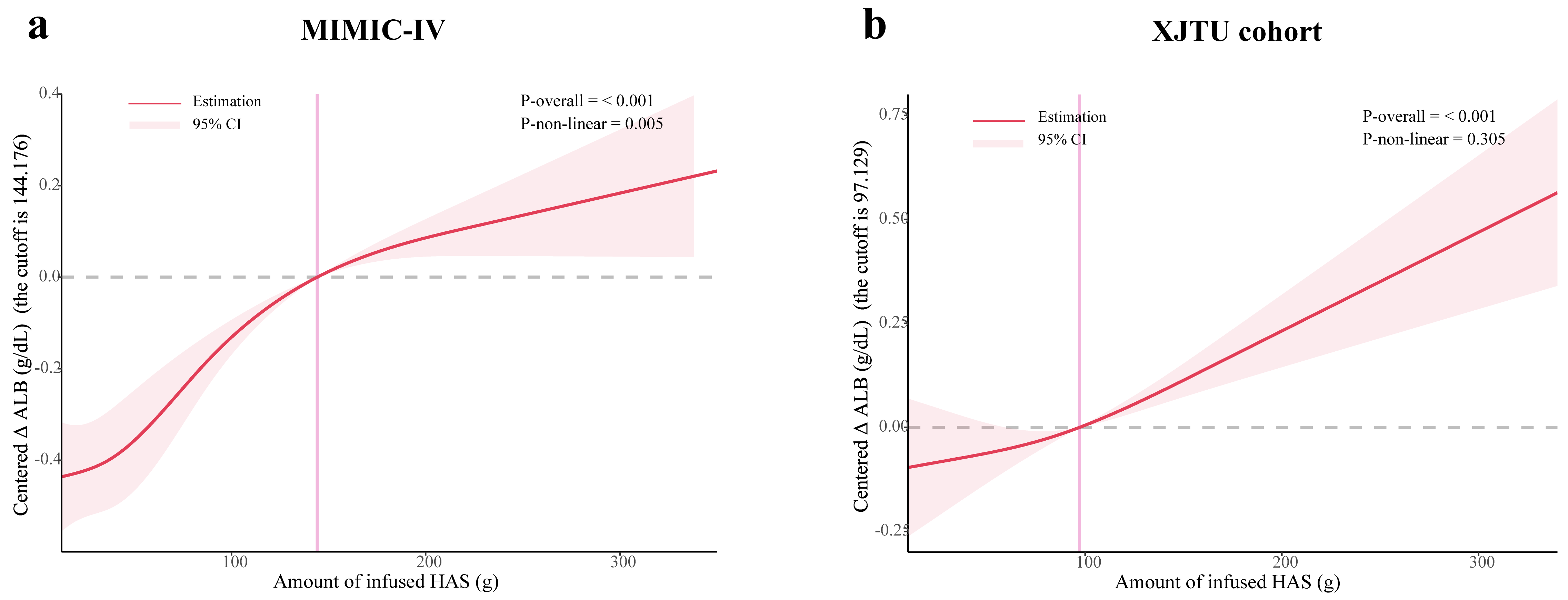


**Figure S5:** Association between albumin dose administered during the first 3 ICU days and ΔALB in patients receiving HAS, based on multivariable linear regression analyses.

(a) MIMIC-IV cohort (n=911).

(b) First Affiliated Hospital of Xi'an Jiaotong University cohort (n=670).


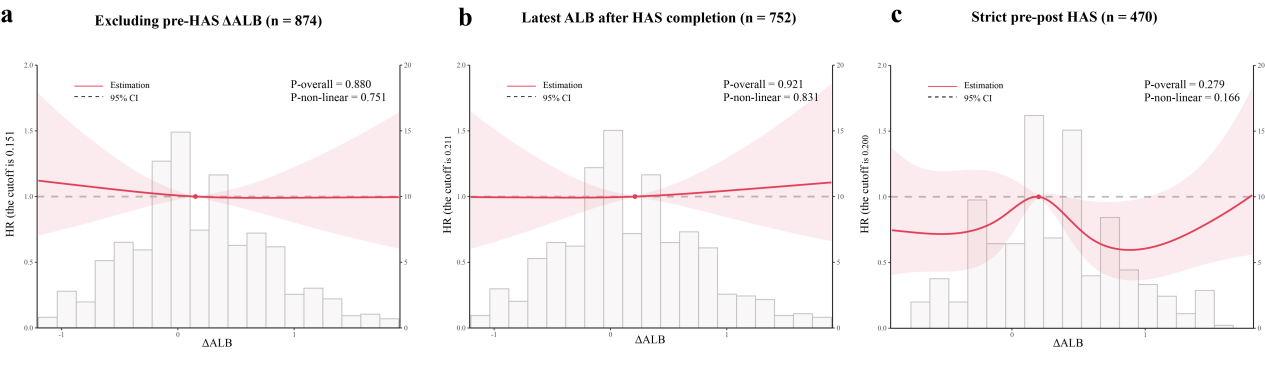


**Figure S6:** Association between HAS-exposed ΔALB and 90-day outcome in time-aligned sensitivity cohorts from MIMIC-IV.

(a) Excluding patients whose ΔALB measurement interval occurred entirely before the first HAS administration (n = 874).

(b) Restricting to patients whose latest albumin measurement occurred after completion of at least one HAS infusion (n = 752).

(c) Strict pre-post HAS cohort, defined as first albumin measurement before first HAS administration and latest albumin measurement after completion of the first HAS infusion (n = 470).

Restricted cubic spline Cox models were adjusted using the same covariates as in the primary analysis.
